# Temporal Variation and Region- and Sex-Specific Expression of GABA_A_ Receptor Subunits in the Mouse Suprachiasmatic Nucleus

**DOI:** 10.3390/biology15181671

**Published:** 2026-09-21

**Authors:** Janelle Chong, James F. Cheeseman, Matthew D. M. Pawley, David Cumin, Nicola Ludin, Andrea Kwakowsky, Guy R. Warman

**Affiliations:** 1 Department of Anaesthesiology, School of Medicine, University of Auckland, Private Bag 92019, Auckland 1142, New Zealand; janelle.chong@outlook.com (J.C.); j.cheeseman@auckland.ac.nz (J.F.C.); d.cumin@auckland.ac.nz (D.C.); 2 Department of Engineering, Computer and Mathematical Sciences, Auckland University of Technology, Auckland 1010, New Zealand; matthew.pawley@aut.ac.nz; 3 Department of Psychological Medicine, School of Medicine, University of Auckland, Private Bag 92019, Auckland 1142, New Zealand; n.ludin@auckland.ac.nz; 4 Pharmacology and Therapeutics, School of Medicine, Galway Neuroscience Centre, Ollscoil na Gaillimhe-University of Galway, H91 W5P7 Galway, Ireland; andrea.kwakowsky@universityofgalway.ie

**Keywords:** circadian, GABA, GABA_A_ receptor, light, suprachiasmatic nucleus

## Abstract

GABAergic neurotransmission plays a central role in regulating the mammalian circadian clock. However, the precise contribution of GABA type A receptor (GABA_A_R) subunits to timekeeping within the suprachiasmatic nucleus (SCN) remains incompletely defined. Here, we investigated the spatiotemporal expression patterns of four GABA_A_R subunits (α1, α5, β3, and γ2) in the SCN of male and female C57BL/6 mice. Our aim was to determine whether these subunits exhibit circadian, regional, and sex-specific variation under entrained (LD12:12) and free-running (DD) conditions. Fluorescence immunohistochemistry revealed the expression of all four subunits in the SCN, with several displaying rhythmic profiles. The α1 subunit peaked during the day in both sexes and was preferentially expressed in the shell region. The α5 subunit also varied across time but lacked strong regional specificity. Temporal variation in β3 and γ2 expression was observed in males but not females, indicating potential sex-dependent regulation. Notably, the γ2 subunit showed a dynamic shift in regional distribution over the circadian cycle, with higher shell expression during the day and core enrichment at night. Under DD conditions, rhythmic expression of the α1 and γ2 subunits persisted, suggesting endogenous control independent of external light cues. However, overall amplitude and regional specificity were reduced in the absence of light. These findings demonstrate that GABA_A_R subunit expression within the SCN is regulated by circadian phase, SCN subregion, and biological sex. This study provides new insight into the molecular architecture of the SCN and supports the hypothesis that subunit-specific GABAergic signalling contributes to the temporal organisation of the circadian clock. Further investigation into the functional significance of these subunit patterns will help clarify their roles in SCN synchrony, photic entrainment, and sex-specific circadian regulation.

## Simple Summary

The circadian clock in the suprachiasmatic nucleus (SCN) of the brain controls daily cycles of sleep, activity, and many other biological processes. A key chemical messenger involved in regulating this clock is gamma-aminobutyric acid (GABA), which helps nerve cells communicate. How different forms of the receptors that respond to GABA contribute to keeping time in the brain’s master clock is poorly understood. In this study, we examined the expression of four GABA receptors in male and female mice and measured how their levels changed throughout the day and night under normal light–dark conditions and in constant darkness. We found that all four receptor components were present in the master clock, and several showed clear daily patterns of expression. Some changes differed between males and females, while others varied between distinct regions of the SCN. Two receptor components continued to show daily rhythms even in constant darkness, indicating that their activity is controlled by the internal clock rather than by external light. These findings improve our understanding of how the brain’s master clock is organised and regulated. This knowledge may help explain differences in biological timing between individuals and could contribute to future research on circadian rhythms.

## 1. Introduction

The suprachiasmatic nucleus (SCN), located in the anterior hypothalamus, is the principal circadian pacemaker in mammals [1,2]. It synchronises daily physiological and behavioural rhythms by coordinating intrinsic cellular oscillators across the brain and peripheral tissues. These rhythms are governed by a well-characterised molecular clock composed of transcriptional–translational feedback loops involving core clock genes such as *Period (Per)*, *Cryptochrome (Cry)*, *Clock*, and *Bmal1* [3,4,5]. While this molecular framework has been extensively studied, the neurochemical mechanisms that underlie synchrony and phase-shifting within the SCN network remain less well understood.

A central component of SCN neurochemistry is γ-aminobutyric acid (GABA), the main inhibitory neurotransmitter in the central nervous system and the predominant neurotransmitter within the SCN [6,7,8]. GABAergic signalling is implicated in key circadian processes including phase resetting, photic entrainment, and intercellular oscillator coupling—functions critical for maintaining coherent and adaptable circadian rhythms [9,10,11,12,13]. These effects appear to be mediated, at least in part, through GABA type A receptors (GABA_A_Rs), which are pentameric chloride channels assembled from combinations of at least 19 known subunit isoforms [14,15]. The subunit composition of GABA_A_Rs determines receptor kinetics, synaptic or extrasynaptic localisation, and sensitivity to pharmacological modulation, thereby shaping the inhibitory tone and temporal precision of neural networks [16,17,18,19].

Multiple GABA_A_R subunits—including α1, α5, β3, and γ2—have been identified in the SCN. There is, however, conflicting evidence regarding mRNA and protein expression levels, with the latter directly determining the active configurations of GABA_A_Rs in the central circadian clock [20,21,22,23,24]. There is also emerging evidence suggesting that GABA_A_R expression within the SCN is neither spatially uniform nor temporally static. Instead, it appears to be regionally heterogeneous, temporally regulated, and potentially influenced by environmental cues [25,26,27].

The SCN itself is anatomically and functionally compartmentalised into a ventrolateral “core” and a dorsomedial “shell.” The core receives direct retinal input and drives rapid light-induced phase shifts, while the shell, although not directly retinorecipient, integrates signals from the core and helps coordinate outputs to peripheral oscillators [28,29,30]. Neurons in the shell remain rhythmic when isolated but exhibit diminished synchrony in the absence of core input [31]. This structural and functional segregation raises the possibility that GABA_A_R subunits may be differentially expressed across these regions, contributing to distinct roles of the core and the shell in circadian timing. Region-specific expression of GABA_A_R subunits within the SCN has been reported in rodent models, with differences noted along dorsomedial and rostrocaudal axes and across the circadian cycle [7,23,32]. However, despite these findings, a comprehensive spatial characterisation of GABA_A_R subunit expression is lacking.

A further, historically underexplored dimension of SCN organisation is biological sex. Circadian studies have traditionally relied heavily on male animals to avoid variability attributed to the female oestrous cycle. As a result, sex differences in circadian systems have been underexamined, despite growing evidence that males and females differ in SCN morphology, firing rate, and responses to phase-shifting paradigms [33,34,35]. Whether these differences extend to GABAergic architecture within the SCN is unknown. Gonadal hormone receptors are expressed in the SCN and brain regions that project to the SCN, and have been shown to influence circadian function [36,37]. Additionally, sex-specific differences in GABA_A_R subunit expression have been reported in other brain regions. Therefore, sex-specific patterns of GABA_A_R expression in the SCN are plausible and may contribute to behavioural and physiological differences between sexes in circadian regulation [38,39,40].

To address these gaps, in the present study, we have systematically investigated the expression of four GABA_A_R subunits—α1, α5, β3, and γ2—in the SCN of adult male and female C57BL/6 mice. Using high-resolution fluorescence immunohistochemistry, we map subunit distribution along the rostrocaudal axis of the SCN, focusing on the ventrolateral core and dorsomedial shell under both light–dark (LD 12:12) and constant darkness (DD) conditions. We also assess whether expression varies with circadian phase and biological sex. Through this approach, we aim to provide a comprehensive analysis of the spatial, temporal, and sex-specific heterogeneity of GABA_A_R subunit expression in the SCN, offering insights into how GABAergic signalling contributes to circadian network organisation and plasticity.

## 2. Materials and Methods

### 2.1. Animals and Housing

All experimental procedures were approved by the University of Auckland Animal Ethics Committee (approval #002138 and #23441) and conducted in accordance with institutional guidelines for the care and use of animals in research. Male and female C57BL/6 mice (8–12 weeks old) were supplied by the Vernon Jansen Unit, University of Auckland. Male and female mice were individually housed in standard transparent cages (Coulbourn Instruments, Allentown, MA, USA) equipped with a rodent running wheel connected to an infrared microswitch (Actimetrics, Wilmette, IL, USA). Animals had *ad libitum* access to food and water throughout the experiment. Cages were maintained in ventilated, sound-attenuated, temperature-controlled environmental chambers (23 ± 1 °C).

### 2.2. Behavioural Analysis

Locomotor wheel-running activity was continuously recorded using the ClockLab data acquisition system (Actimetrics, Wilmette, IL, USA). Wheel-running locomotor activity data were collected as revolutions or counts per minute through the IR sensor and transferred to ClockLab Analysis software CLA6 (ClockLab; Actimetrics, Wilmette, IL, USA), where data were transformed into actograms. The onset of wheel-running activity (defined as CT12) was determined from the actograms in ClockLab. This onset of activity served as the phase reference point for calculating free-running periods (τ) in constant conditions (DD). For experiments in which animals were maintained in light cycles, results are presented with respect to Zeitgeber time (ZT), with ZT0 defined as lights-on and ZT12 as lights-off.

### 2.3. Tissue Collection

Following habituation, mice were either maintained under a 12:12 h light–dark (LD) cycle or transferred to constant darkness (DD). After 14 days in their respective lighting conditions, animals were euthanised via subcutaneous injection of ketamine (75 mg/kg) and medetomidine (1 mg/kg), followed by transcardial perfusion with 25 mL of ice-cold 4% paraformaldehyde (PFA) in phosphate buffer (pH 7.6). Brains were immediately extracted, post-fixed in 4% PFA for 2 h at room temperature, and subsequently cryoprotected overnight in 30% sucrose prepared in Tris-buffered saline (TBS, pH 7.6) at 4 °C. Serial coronal brain sections (30 µm thick) were cut using a freezing microtome and stored in cryoprotectant solution at –20 °C. Tissues were collected at Zeitgeber times (ZT) 0, 6, 12, and 18 from mice housed under LD cycles, and at circadian times (CT) 0, 6, 12, and 18 from mice maintained in DD. ZT12 corresponds to activity onset in nocturnal animals. For all DD collections, brains were harvested under dim red light (<5 lux) to minimise light-induced effects.

### 2.4. Immunohistochemistry

Free-floating fluorescence immunohistochemistry was conducted to detect GABA_A_R α1, α5, β3, and γ2 subunits in the mouse SCN, as described previously [41]. The specificity of primary antibodies was validated by Western blotting in prior studies [42,43,44]. Tissue sections were first incubated for 1 h at room temperature in blocking solution containing 1% donkey serum in 0.05 M tris-buffered saline (TBS) with 0.3% Triton X-100 and 0.25% bovine serum albumin (TTB). After three 10 min washes in TBS, sections were incubated with the respective primary antibodies diluted in TTB: α1 (rabbit anti-α1, Alomone Laboratories, Jerusalem, Israel, 1:1000), α5 (rabbit anti-α5, ThermoFisher Scientific, Waltham, MA, USA, 1:1000), β3 (mouse anti-β3, Novus, 1:500), and γ2 (rabbit anti-γ2, Synaptic Systems, Göttengen, Germany, 1:1000) for 48 h at 4 °C. For regional analysis of GABA_A_R subunits, tissue sections were co-stained with arginine vasopressin (AVP; guinea pig anti-AVP, Synaptic Systems, 1:500). Following primary antibody incubation, sections were washed and incubated for 1 h at room temperature with the appropriate Alexa Fluor-conjugated secondary antibodies (1:500): donkey anti-rabbit Alexa Fluor 647 for α1, α5, and γ2 (Invitrogen, Waltham, MA, USA), donkey anti-mouse Alexa Fluor 488 for β3 (Invitrogen, Waltham, MA, USA), and donkey anti-guinea pig Alexa 594 for AVP (Invitrogen, Waltham, MA, USA). Cell nuclei were counterstained with Hoechst 33342 (Hoechst AG, Frankfurt, Germany) (1:10,000) for 30 min to delineate the SCN boundaries. Sections were mounted on slides, air-dried overnight at room temperature, and coverslipped with Mowiol mounting medium (Hoechst AG, Frankfurt, Germany). Negative controls omitting the primary antibody were included in each run and consistently showed a complete absence of immunoreactivity. Two sequential sets of tissue sections were combined for each GABA_A_R marker immunolabeling to account for mechanical damage to the sections. Further, IHC runs were coordinated to process all tissue sections for each protein of interest simultaneously, enabling direct comparisons of relative protein levels across different groups; e.g., for analysis of the α1 subunit in males under DD conditions, tissue from all four timepoints (CT0, CT6, CT12, CT18) were simultaneously immunolabeled for α1.

### 2.5. Imaging and Analysis

For quantification, imaging was performed using a Zeiss LSM 710 inverted confocal laser-scanning microscope (Carl Zeiss, Jena, Germany) equipped with a Plan-Apochromat 20×/0.8 NA objective (Carl Zeiss, Jena, Germany). SCN boundaries were identified using Hoechst nuclear staining. Receptor density measurements were conducted using ImageJ (version 1, 50I, National Institutes of Health, Bethesda, MD, USA). Prior to analysis, images underwent background subtraction to correct for uneven illumination and grayscale thresholding to eliminate saturated pixels. GABA_A_R subunit densities were quantified for each unilateral SCN and then averaged to generate a single bilateral density value per section. These values were normalised to SCN area to calculate an integrated SCN density. A minimum of three sections per animal were analysed, and the resulting values were averaged within treatment groups. To control for variability across staining runs, integrated densities were normalised to the range of values within each immunohistochemistry batch for each GABA_A_R subunit. All image acquisition and analysis were performed by an experimenter blinded to treatment group to minimise bias.

### 2.6. Statistical Analysis

Sex-specific differences in GABA_A_R subunit staining intensity were assessed using analysis of variance (ANOVA), followed by Tukey’s Honestly Significant Difference (HSD) post hoc test for pairwise comparisons. For regional analysis of each GABA_A_R subunit, signal density values in the SCN under DD were normalised to the combined range of core and shell values from male mice. This normalisation was also applied to values obtained under LD conditions, separately for males and females. To compare subunit expression between the core and shell SCN regions at each circadian timepoint (CT), data were analysed using two-way ANOVA followed by Bonferroni’s multiple comparisons test. Changes in subunit expression across CTs within the core or shell were analysed separately using two-way ANOVA with Tukey’s HSD post hoc test. All statistical analyses were conducted using GraphPad Prism version 11.0.0 for Windows, GraphPad Software, Boston, MA, USA, www.graphpad.com accessed 20 March 2026. F values are shown in Supplementary S2 Table S1.

## 3. Results

### 3.1. Behavioural Results

There were 154 mice used in this study. Of those, 43 males and 64 females were maintained in light cycles (LD 12:12), and 47 males were kept in constant darkness prior to sacrifice and subsequent analysis of GABA_A_R subunit expression. There were a total of 1469 sections analysed. All animals displayed clearly entrained nocturnal behavioural rhythms under light–dark cycles (LD12:12) and an average free-running period of 23.68 h (±0.04 h) in constant conditions (DD) (individual actograms and free-running periods supplied in Supplementary S1, Figure S1; Supplementary S2, Table S5).

### 3.2. Diel- and Sex-Dependent Expression of GABA_A_R Subunits in the SCN

**Figure 1:**
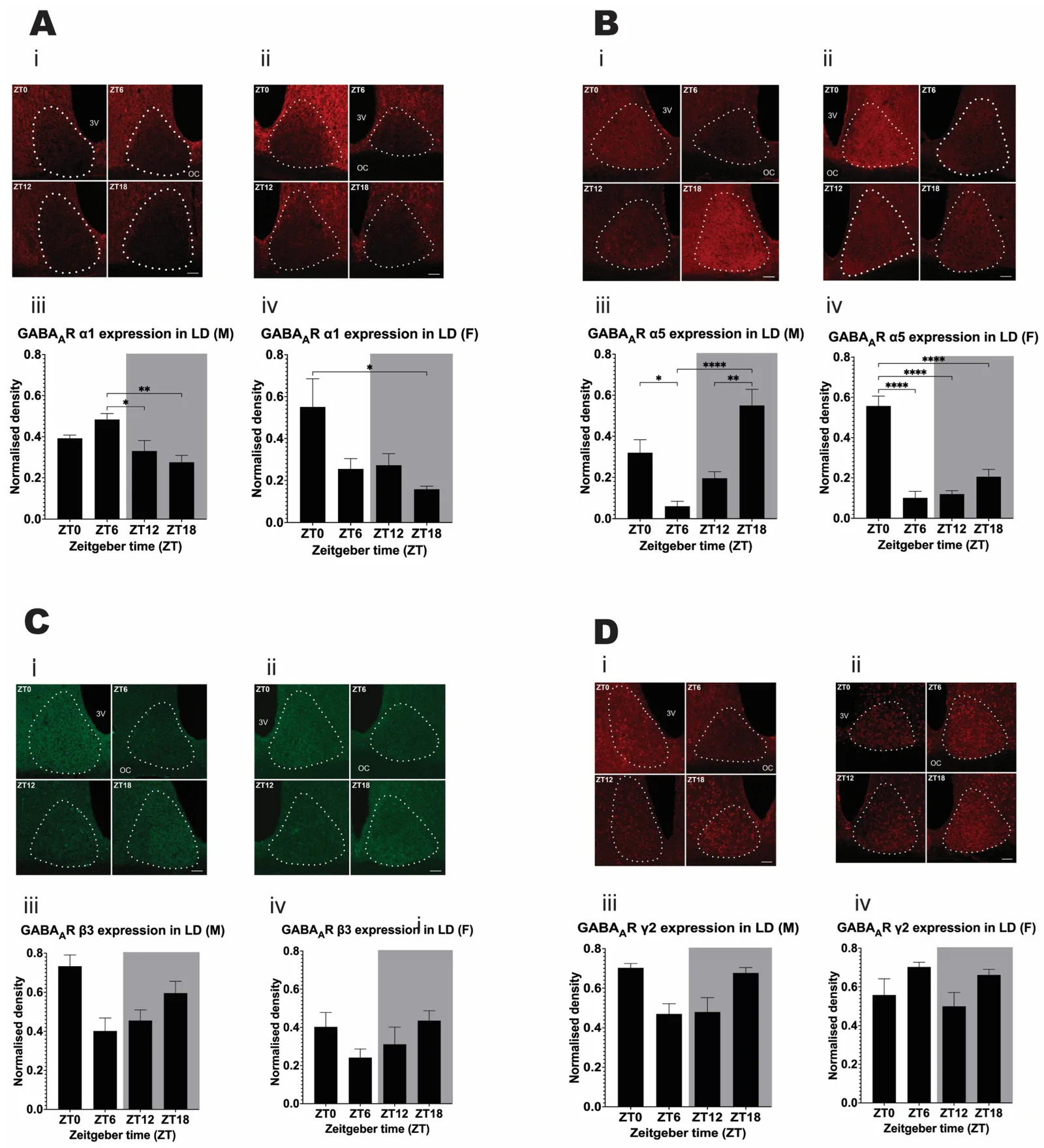
Expression patterns of four GABA_A_R subunits (α1, α5, β3, and γ2) in the suprachiasmatic nuclei of C57 mice maintained in light cycles (LD 12:12). (**A**) Expression of α1 subunit. (**i**) Representative sections in male mice and (**iii**) bar graph of these expression levels over 24 h with statistically significant differences denoted by asterisks (ANOVA); one asterisk *p* < 0.05, two asterisks *p* < 0.01, four asterisks *p* < 0.0001. (**ii**) Representative sections in female mice and (**iv**) bar graphs of these expression levels over 24 h with statistically significant differences denoted by asterisks. (**B**–**D**) show data from α5, β3, and γ2, respectively, with (**i**) and (**iii**) representing males and (**ii**) and (**iv**) females.

In agreement with our previous findings on α1, α5, β3, and γ2 in male mice, the immunohistochemical analysis conducted here showed that all four GABA_A_R subunits (α1, α5, β3, and γ2) are expressed in the SCN of mice in light–dark cycles, and that their expression displays distinct patterns which vary both temporally and by sex (Table 1, Figure 1, Supplementary S2, Table S2).

The α1 subunit was predominantly localised to the dorsal shell region of the SCN in both sexes. Quantitative analysis showed significant diel variation in α1 expression in males (*p* = 0.0007), with a clear peak at ZT6 and significant reductions at ZT12 (*p* = 0.0119) and ZT18 (*p* = 0.0005) (Figure 1A). Females also exhibited rhythmic variation (*p* = 0.0291), with a significant decline from the peak of expression at ZT0 until ZT18 (*p* = 0.0409) (Figure 1A).

In contrast, the α5 subunit displayed a more diffuse expression across the SCN neuropil and moderate cell body staining in both sexes. Time of day significantly influenced α5 protein expression in both males (Figure 1B) (*p* < 0.0001) and females (Figure 1B) (*p* < 0.0001). While α5 protein expression peaked at ZT18 in males, it peaked at ZT 0 in females. Despite differences in peak expression, both sexes exhibited similar overall rhythmic profiles, with troughs occurring at ZT6 and ZT12 (Figure 1B).

The β3 subunit was also diffusely expressed throughout the SCN. In males, β3 protein expression showed a significant diel pattern (Figure 1C) (*p* = 0.0364), with a peak and nadir at ZT0 and ZT6, respectively. While female mice showed a similar temporal pattern, this was not statistically significant (Figure 1C).

The γ2 subunit exhibited a daily rhythm in expression exclusively in males, with a similar temporal pattern to α5 and β3, peaking at ZT0 and ZT18 (Figure 1D) (*p* = 0.0120). Females showed no consistent time of day variation in γ2 expression across timepoints (Figure 1D).

### 3.3. Regional Expression of GABA_A_R Subunits in the SCN Under LD Conditions

**Figure 2:**
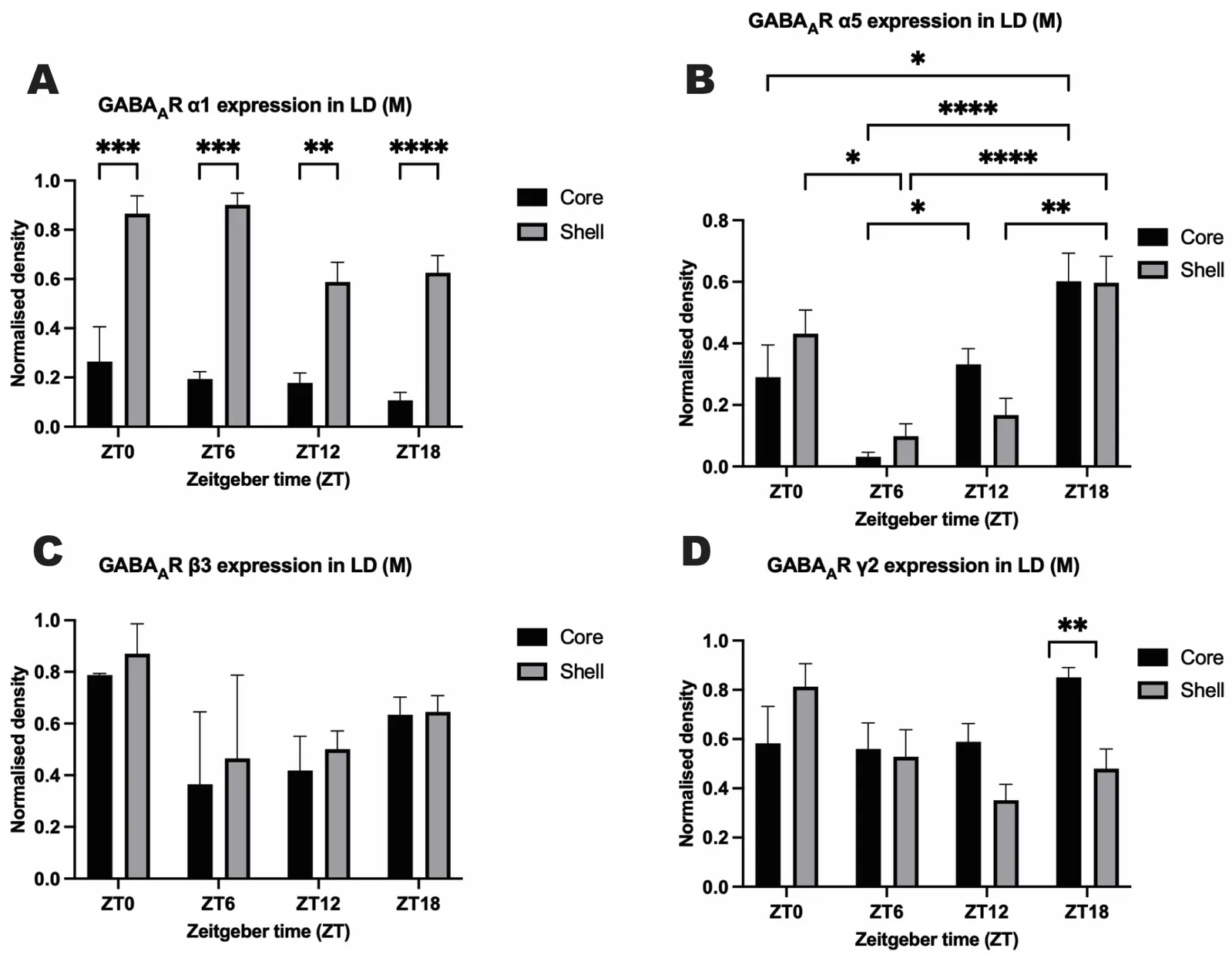
Bar graphs of the regional expression patterns of four GABA_A_R subunits (α1, α5, β3, and γ2) in the suprachiasmatic nuclei of male C57 mice maintained in light cycles (LD 12:12). (**A**) α1 subunit; (**B**) α5 subunit; (**C**) β3 subunit; and (**D**) γ2 subunit. Statistically significant differences denoted by asterisks (ANOVA); one asterisk *p* < 0.05, two asterisks *p* < 0.01, three asterisks *p* < 0.001, four asterisks *p* < 0.0001.

**Figure 3:**
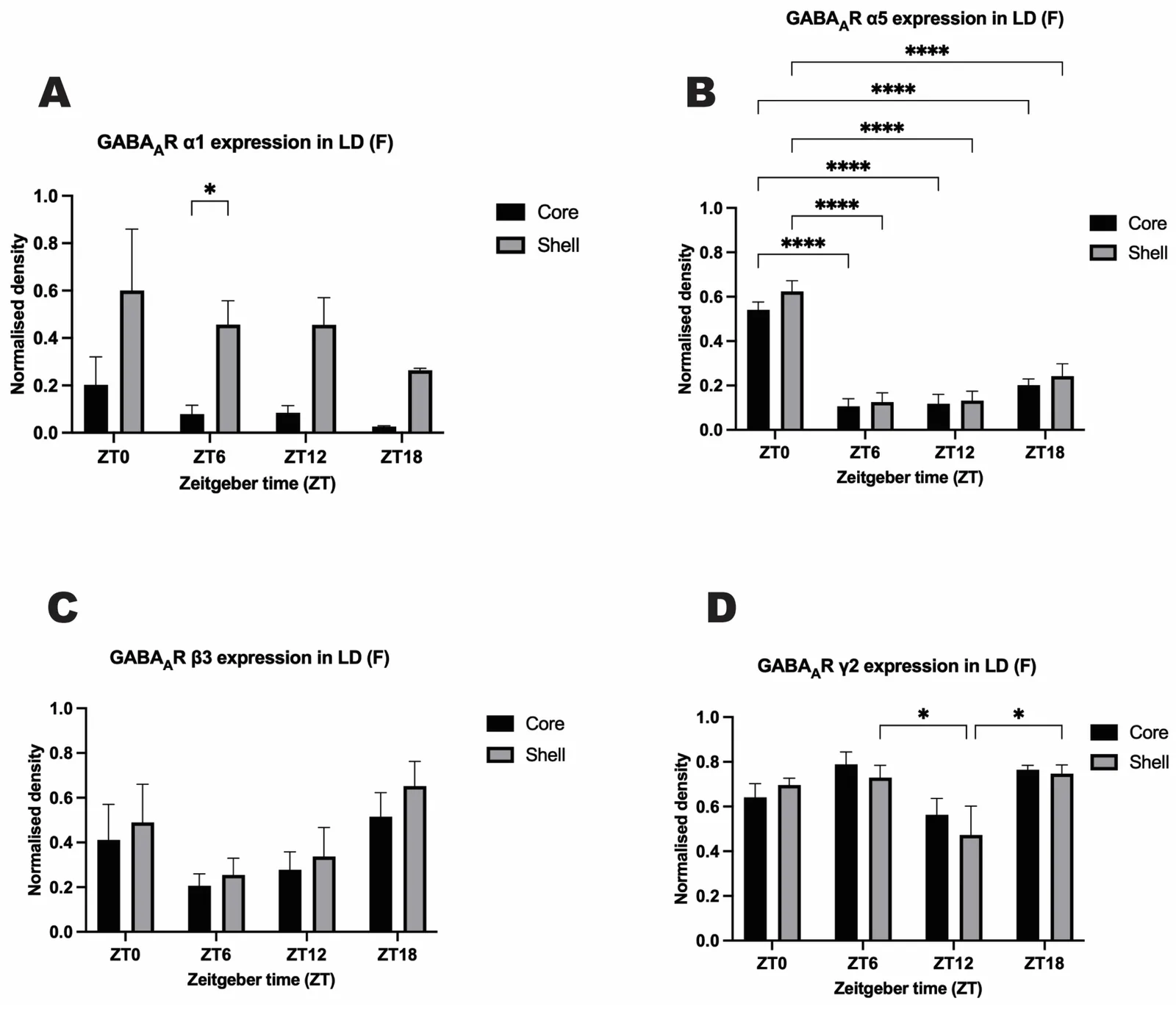
Bar graphs of the regional expression patterns of four GABA_A_R subunits (α1, α5, β3, and γ2) in the suprachiasmatic nuclei of female C57 mice maintained in light cycles (LD 12:12). (**A**) α1 subunit; (**B**) α5 subunit; (**C**) β3 subunit; and (**D**) γ2 subunit. Statistically significant differences denoted by asterisks (ANOVA); one asterisk *p* < 0.05, four asterisks *p* < 0.0001.

Given the different functional roles of the retinorecipient core region of the SCN and its shell, an understanding of the relative expression of the different subtypes of GABA_A_R subunits in each should provide insight into understanding their potential roles in entrainment versus rhythm generation. Thus, we examined the expression patterns of α1, α5, β3, and γ2 from 110 male and 144 female SCNs over the course of 24 h (Table 2, Figure 2 and Figure 3, Supplementary S2, Tables S2 and S3).

The overarching finding here was that, consistent with its known dorsal localisation, the α1 subunit was primarily expressed in the shell region of the SCN in both males and females (Figure 2A and Figure 3A) (*p* < 0.0001 for males; *p* = 0.0001 for females), whereas the other subunits showed similar expression levels in both the core and the shell regions (Figure 2 and Figure 3B–D).

Alpha 1 exhibited relatively consistent expression across 24 h in the core region of the SCN but showed a clear diel expression pattern in the shell of males peaking at ZT 0-6 (Figure 2A) (*p* = 0.0254). While females showed a trend towards higher expression in the shell at ZT0, there was no statistically significant time of day rhythm in expression (*p* = 0.2113).

By contrast, the α5 subunit was similarly expressed throughout the SCN, showing no significant regional variation in either sex (Figure 2 and Figure 3B) (*p* = 0.2492 for males; *p* = 0.2023 for females), but strong diel variations were evident in both sexes (Figure 2 and Figure 3B) and particularly prominent in females, whose expression peaked strongly at ZT0 and was low at all other times (*p* < 0.0001 for both sexes).

The β3 subunit also lacked region-specific variation, with no significant differences observed between the core and shell in either sex. However, diel variation was again present, with statistically significant peaks in expression in males at ZT 0 and ZT18 (*p* = 0.0324). There was no similar significant difference in females (*p* = 0.171).

The γ2 subunit had less clear patterns of expression than the other subunits. In males, there was a potential antiphase expression of γ2 in the two regions with significant interaction between ZT and the SCN region (*p* = 0.0072), indicating higher expression in the shell during the day and in the core at night. In females, however, there were no region differences (*p* = 0.5201).

### 3.4. Expression of GABAAR Subunits in the SCN Under DD Conditions

**Figure 4:**
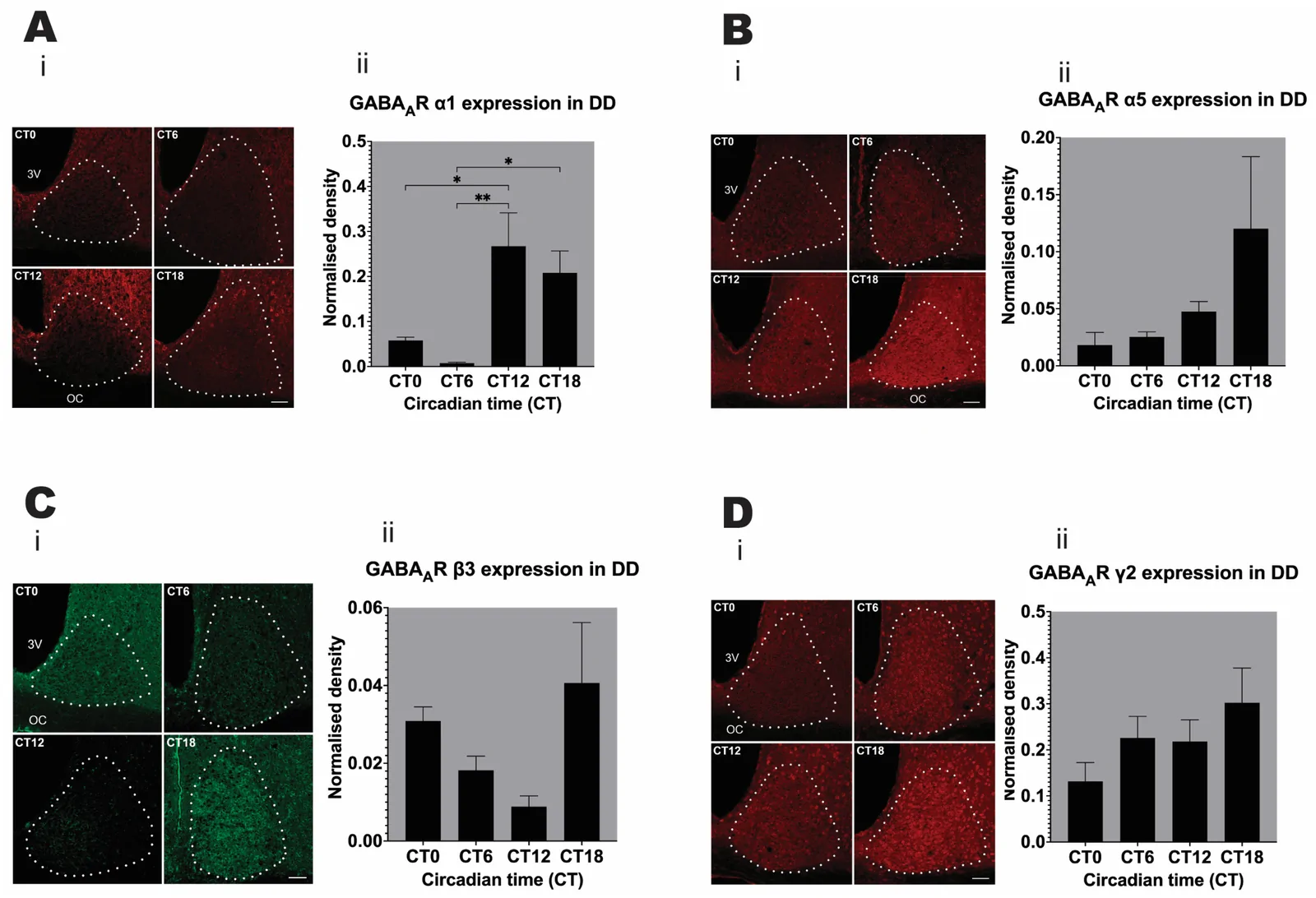
Expression patterns of four GABA_A_R subunits (α1, α5, β3, and γ2) in the suprachiasmatic nuclei of male C57 mice maintained in constant conditions (DD). (**A**): Expression of α1 subunit. (**i**) Representative sections and (**ii**) bar graph of these expression levels over 24 h, with statistically significant differences denoted by asterisks (ANOVA); one asterisk *p* < 0.05, two asterisks *p* < 0.01; (**B**–**D**) show data from α5, β3, and γ2 respectively.

The diel variations in expression of the different GABA_A_R subunits in light cycles could result either from a response to ambient light cycles or from an endogenously driven circadian rhythm in expression. In order to differentiate between these possibilities, we conducted expression analysis of α1, α5, β3, and γ2 receptor subtypes in male mice maintained in constant conditions (DD) for 14 days (Table 3, Figure 4, Supplementary S2, Table S4).

Under constant darkness (DD), analysis of GABA_A_R subunit expression in male mice revealed subunit-specific differences in spatial and temporal regulation across the SCN (Figure 4). The α1 subunit was the only receptor that exhibited robust circadian variation, with expression peaking at CT12 (Figure 4). While there appeared to be a trend towards rhythmicity in expression in α5, β3, and γ2, these rhythms were of very low amplitude (particularly for α5, β3), were not statistically significant, and are almost certainly of little biological relevance (Table 3).

The significantly damped expression of α5, β3, and γ2 is unlikely to reflect differences in phase of the different individuals used for sampling, as the variation in the free-running period of these mice was 0.04 h (or 2.4 min). Thus, after 14 days, in DD, the variability in phase between different mice is unlikely to be more than 33 min (which is well within the 6 h time bins in which data were analysed). Thus, it can be reasonably assumed that the rhythms in the expression of α5, β3, and γ2 are primarily driven by the light–dark cycle, but the rhythms of α1 expression may be driven, at least in part, by endogenous mechanisms.

## 4. Discussion

This study provides a comprehensive examination of the spatiotemporal sex- and region-specific expression patterns of four GABA_A_R subunits—α1, α5, β3, and γ2—within the mouse SCN under both LD and DD conditions. The findings confirm that GABA_A_R subunit expression in the SCN is dynamic, varies across the circadian cycle, differs between SCN subregions, and is modulated by biological sex. These results provide new molecular insight into how GABAergic signalling may contribute to the complex spatiotemporal architecture of the mammalian central clock.

Among the subunits studied, α1 and γ2 displayed the most robust circadian rhythmicity and regional specificity, particularly under LD conditions. Expression of the α1 subunit was consistently enriched in the SCN shell and peaked during the subjective day in both sexes—a pattern preserved, albeit dampened, under DD conditions. Fast, phasic inhibition in the brain is mediated by distinct GABA_A_R subtypes, with α1-containing receptors being the most prevalent [18,45,46]. In the SCN, this form of synaptic inhibition is thought to contribute to the precise timing of neuronal firing, including the phase-shifting and synchronisation of clock cells [12,13,26]. These results are consistent with previous work from our group showing similar temporal dynamics of expression of these receptor subtypes in male C57 mice [25]. The concordance between these studies strengthens the evidence that temporal regulation of these GABA_A_R subunits is present in the SCN and may reflect an intrinsic feature of SCN GABAergic signalling. Previous studies have also found a rhythmic balance of expression between the ratio of phasic-mediating GABA_A_Rs (via the γ2 subunit) and tonic-mediating GABA_A_Rs (via the δ subunit) in the SCN [32]. Their results showed a greater abundance of GABA_A_-phasic receptors in the SCN during the subjective day, consistent with our observations of the α1 subunit, and greater abundance of GABA_A_-tonic receptors during the subjective night. These findings suggest that elevated daytime expression of α1-containing GABA_A_Rs may enhance phasic inhibition in the SCN shell, supporting time-of-day-dependent coordination of neuronal activity that underlies coherent circadian output.

GABA_A_Rs containing the α5 subunit are primarily involved in mediating sustained, tonic inhibition [47,48]. Recent findings have highlighted an expanded role for tonic GABAergic signalling in the SCN, including its involvement in regulating intracellular chloride concentration across the circadian cycle and influencing daily rhythms in neuronal excitability and behaviour [49,50,51,52]. Although our results showed no regional preference in α5 subunit protein expression and revealed broad temporal fluctuations across the SCN neuropil, the subunit nonetheless appears to be under circadian regulation. Its diffuse distribution and lack of spatial specificity may indicate a more generalised role in modulating extrasynaptic, tonic inhibition throughout the SCN. This widespread and spatially non-specific expression pattern may reflect a more generalised role in modulating tonic inhibition throughout the SCN, which, in turn, could contribute to setting the baseline excitability of SCN neurons and shaping daily rhythms in neuronal activity and behaviour.

Interestingly, β3 and γ2 subunit expression demonstrated temporal variation that was primarily restricted to male mice, with females showing more static expression profiles. The γ2 subunit, in particular, exhibited a striking diurnal redistribution, shifting from shell-dominant expression during the day to core enrichment at night. This dynamic spatial shift suggests a role for γ2-containing receptors in mediating region-specific network plasticity and possibly photic responsiveness, particularly in males. One possibility is that γ2 relocalisation shifts inhibitory tone toward the core at night, when the SCN is most responsive to light, thereby enhancing the precision of photic entrainment through region-specific GABAergic gating. These findings build on previous work in hamsters [32] and extend it by demonstrating that such patterns are not only circadian but also sexually dimorphic. Our findings are broadly consistent with the time-dependent regulation of γ2 expression reported by Walton et al. [32], although the timing of maximal expression differs between studies. Walton et al. observed higher γ2 expression at ZT13 and ZT19 relative to ZT6, whereas in the present study, expression peaked at comparable levels at ZT0 and ZT18 and showed comparable nadirs at ZT6 and ZT12. Thus, both studies support circadian variation in γ2 expression but differ in the apparent phase of this variation. This discrepancy may reflect differences in experimental design, including the use of four sampling time points in the present study compared with three in Walton et al., as well as differences in animal models, as Walton et al. used Syrian hamsters while the present study used C57BL/6 mice. Differences in sampling resolution or species-specific regulation may, therefore, contribute to the distinct temporal profiles observed.

Under free-running conditions, the expression of both α1 and γ2 subunits remained rhythmic, although with reduced amplitude and regional specificity. This suggests that light refines and amplifies the spatial organisation of GABA_A_R expression, particularly in the shell and core. The reduced distinction between regions under DD—especially for γ2—may reflect diminished photic entrainment and a blunting of SCN subregion specialisation in the absence of external cues. These findings support the idea that light is not merely an entraining signal but also shapes the neurochemical architecture of the SCN in a spatially heterogeneous manner. The β3 expression retained circadian rhythmicity under DD, peaking during the late subjective night (CT18), suggesting its regulation is at least partially independent of environmental light. Its lack of regional specificity across lighting conditions implies a more generalised role of β3-containing GABA_A_Rs in maintaining basal inhibitory tone throughout the SCN, contrasting with the compartmentalised dynamics observed for α1 and γ2.

Sex-specific patterns of GABA_A_R expression were observed across multiple subunits, with males exhibiting more pronounced temporal regulation than females. This was particularly evident for γ2, which showed robust time- and region-dependent modulation in males but remained largely invariant in females. These findings may reflect the influence of the oestrous cycle on circadian rhythmicity through changes in gonadal hormone levels. Because the stage of the oestrous cycle was not controlled or accounted for in the present study, variation in cycle stage among females may have contributed to differences in the phase and magnitude of receptor expression and thereby attenuated temporal variation at the group level. These molecular distinctions echo previously reported sex differences in SCN neuronal firing rate, morphology, and hormone receptor distribution [35]. They may underlie sex-specific variations in circadian behaviour, such as shorter free-running periods and enhanced photic sensitivity in females, possibly through differences in how GABAergic inhibition modulates neuronal synchrony or signal propagation within the SCN [53]. Additionally, the phase opposition in α5 expression between sexes—with males peaking at ZT18 and females at ZT0—suggests differential regulation of tonic inhibition that could affect sex-dependent SCN excitability and behavioural output. Such divergence may have implications for chronotype differences, phase resetting thresholds, or susceptibility to circadian disruption. These findings underscore the importance of including sex as a biological variable in circadian neurobiology and call for future work to determine whether these expression differences reflect hormonal influences, chromosomal effects, or both.

The observed diversity in GABA_A_R subunit expression patterns—across time, region, and sex—highlights the functional heterogeneity of GABAergic signalling within the SCN. Subunit-specific temporal dynamics likely underlie different synaptic and extrasynaptic inhibitory mechanisms that modulate excitability, synchrony, and entrainment across core and shell regions. The shifting distribution of γ2 from shell to core, in particular, may represent a mechanism through which GABAergic tone is redistributed to enhance photic responsiveness at night. It may also facilitate transitions between synchronised and desynchronised SCN states, depending on internal physiological or external environmental demands. Together, our findings support the emerging view that GABA’s role in the SCN is highly context-dependent, varying by subunit composition, cellular localisation, and circadian phase [26,27].

## 5. Limitations

While this study provides high-resolution mapping of four GABA_A_R subunits, it does not capture the full diversity of receptor compositions possible within the SCN. Despite mediating a distinct form of GABAgeric signalling, the extrasynaptic δ subunit is considered important in maintaining circadian rhythm robustness and intracellular synchrony in the SCN. Emerging evidence further suggests that there may be substantial functional redundancy between the γ2 and δ subunits, as overexpression of either γ2 or δ can compensate for loss of the other [52]. Consequently, the γ2 expression patterns identified here cannot be interpreted in isolation as reflecting the full temporal regulation of GABA_A_R-mediated inhibition within the SCN. Extending the present analysis to δ subunit expression would allow the determination of whether δ exhibits a complementary, opposing, or coordinated temporal pattern relative to γ2, and would, therefore, help establish whether the observed γ2 rhythms reflect selective changes in phasic receptor composition or broader circadian remodelling of GABA_A_R populations. Future investigations should incorporate additional subunits (e.g., δ, ε, θ) and combine protein expression with functional assays such as patch-clamp recordings and pharmacological blockade. For example, selective inhibition of α5-containing receptors could clarify their role in maintaining daytime SCN excitability, while chemogenetic activation of GABAergic neurons in core versus shell regions could help determine how γ2 redistribution contributes to region-specific entrainment, particularly in males versus females. One important limitation of the work described here is that the phase of the oestrous cycle of female mice was neither controlled nor accounted for. This may have contributed, at least in part, to the differences observed in the expression of receptors between the sexes. Controlling for oestrous phase, hormonal manipulations (e.g., gonadectomy, hormone replacement) and genetic approaches (e.g., SCN-specific knockouts) will also be key to disentangling the mechanisms underlying sex differences in subunit expression.

## 6. Conclusions

In conclusion, this study demonstrates that GABAAR subunit expression within the SCN is regulated in a region-specific, time-of-day-dependent, and sex-dependent manner, revealing a previously under-defined level of complexity in the SCN’s inhibitory network. The α1 and γ2 subunits show dynamic rhythmic expression patterns that are both light-responsive and endogenous, while sex differences in β3 and γ2 expression suggest distinct mechanisms of GABAergic modulation between males and females. These findings highlight the importance of considering both spatial compartmentalisation and biological sex in studies of SCN function. More broadly, they provide a strong rationale for future work aimed at uncovering how subunit-specific GABAergic signalling contributes to the temporal organisation, plasticity, and robustness of the mammalian circadian clock.

## Figures and Tables

**Table 1:** Quantification of expression of four GABA_A_R subunits (α1, α5, β3, and γ2) in the suprachiasmatic nuclei of C57 mice maintained in light cycles (LD 12:12). Values are shown as mean normalized density (standard error). Sample sizes for timepoints ZT 0, 6, 12 and 18 for α1 for males were 5, 6, 5, and 6, respectively, and 5, 6, 6 and 3 for females; for α5, they were 4, 5, 5, and 6 for males and were 5, 5, 6 and 5 for females; for β3, they were 2, 4, 4, and 6 for males and 6, 6, 5 and 6 for females; and for γ2, they were 3, 4, 6 and 6 for males and 6, 6, 5 and 6 for females. Multiple ICC sections (up to 10) were analysed for each animal. Details of number of sections are provided in Supplementary S2, Table S2.

	*Alpha 1*		*Alpha 5*		*Beta 3*		*Gamma 2*	
ZT	Males (n = 22)	Females (n = 20)	Males (n = 20)	Females (n = 21)	Males (n = 16)	Females (n = 23)	Males (n = 19)	Females (n = 23)
0	0.39 (0.01)	0.55 (0.13)	0.32 (0.06)	0.56 (0.05)	0.73 (0.06)	0.40 (0.08)	0.70 (0.02)	0.56 (0.08)
6	0.48 (0.03)	0.25 (0.05)	0.06 (0.02)	0.10 (0.03)	0.40 (0.07)	0.24 (0.05)	0.47 (0.05)	0.70 (0.02)
12	0.33 (0.05)	0.27 (0.06)	0.20 (0.03)	0.12 (0.02)	0.46 (0.05)	0.31 (0.09)	0.48 (0.07)	0.50 (0.07)
18	0.28 (0.03)	0.16 (0.01)	0.55 (0.08)	0.21 (0.04)	0.60 (0.06)	0.44 (0.05)	0.68 (0.03)	0.66 (0.03)

**Table 2:** Quantification of regional expression of four GABA_A_R subunits (α1, α5, β3, and γ2) in the suprachiasmatic nuclei of C57 mice maintained in light cycles (LD12:12). Values are shown as mean normalized density (standard error) in the core or shell of the SCN. Sample sizes for each timepoint are the same as reported in Table 1 and details of section numbers are provided in Supplementary S2, Tables S2 and S3.

	*Alpha 1* (n = 26)		*Alpha 5* (n = 32)		*Beta 3* (n = 26)		*Gamma 2* (n = 26)	
ZT	Males—Core	Males—Shell	Males—Core	Males—Shell	Males—Core	Males—Shell	Males—Core	Males—Shell
0	0.26 (0.14)	0.87 (0.07)	0.29 (0.1)	0.43 (0.80)	0.79 (0.01)	0.87 (0.12)	0.58 (0.15)	0.81 (0.09)
6	0.19 (0.03)	0.90 (0.05)	0.03 (0.02)	0.10 (0.04)	0.36 (0.28)	0.46 (0.32)	0.56 (0.11)	0.53 (0.11)
12	0.18 (0.04)	0.59 (0.08)	0.33 (0.05)	0.17 (0.06)	0.42 (0.13)	0.50 (0.07)	0.59 (0.07)	0.35 (0.06)
18	0.11 (0.03)	0.63 (0.07)	0.60 (0.09)	0.60 (0.09)	0.63 (0.07)	0.65 (0.06)	0.85 (0.04)	0.48 (0.08)
	***Alpha 1* (n = 30)**		***Alpha 5* (n = 38)**		***Beta 3* (n = 38)**		***Gamma 2* (n = 38)**	
**ZT**	**Females—Core**	**Female—Shell**	**Females—Core**	**Females—Shell**	**Females—Core**	**Females—Shell**	**Females—Core**	**Females—Shell**
0	0.20 (0.12)	0.60 (0.26)	0.54 (0.04)	0.62 (0.05)	0.41 (0.16)	0.49 (0.17)	0.64 (0.06)	0.70 (0.03)
6	0.08 (0.04)	0.46 (0.10)	0.11 (0.03)	0.13 (0.04)	0.21 (0.05)	0.25 (0.08)	0.79 (0.06)	0.73 (0.05)
12	0.08 (0.03)	0.46 (0.12)	0.12 (0.04)	0.13 (0.04)	0.28 (0.08)	0.34 (0.13)	0.56 (0.07)	0.47 (0.13)
18	0.03 (0.003)	0.26 (0.01)	0.20 (0.03)	0.24 (0.06)	0.52 (0.11)	0.56 (0.11)	0.76 (0.02)	0.73 (0.04)

**Table 3:** Quantification of regional expression of four GABA_A_R subunits (α1, α5, β3, and γ2) in the suprachiasmatic nuclei of male C57 mice maintained in constant conditions (DD). (**A**) Values are shown as mean normalized density (standard error) in SCN as a whole and (**B**) in the separate core and shell regions of the SCN.

A								
	***Alpha 1***		***Alpha 5***		***Beta 3***		***Gamma 2***	
**CT**	**n = 24**	**Density**	**n = 23**	**Density**	**n = 23**	**Density**	**n = 23**	**Density**
0	6	0.06 (0.01)	5	0.02 (0.01)	5	0.03 (0.004)	5	0.13 (0.04)
6	6	0.01 (0.002)	6	0.03 (0.004)	6	0.02 (0.004)	6	0.23 (0.05)
12	6	0.27 (0.07)	6	0.05 (0.01)	6	0.01 (0.003)	6	0.22 (0.05)
18	6	0.21 (0.05)	6	0.12 (0.06)	6	0.04 (0.02)	6	0.30 (0.08)
B								
	***Alpha 1***		***Alpha 5***		***Beta 3***		***Gamma 2***	
**CT**	**core**	**shell**	**core**	**shell**	**core**	**shell**	**core**	**shell**
0	0.01 (0.002)	0.18 (0.05)	0.08 (0.06)	0.14 (0.01)	0.04 (0.02)	0.01 (0.003)	0.08 (0.03)	0.15 (0.04)
6	0.001 (0.0004)	0.02 (0.01)	0.03 (0.004)	0.03 (0.01)	0.04 (0.01)	0.03 (0.02)	0.15 (0.04)	0.24 (0.04)
12	0.05 (0.02)	0.48 (0.12)	0.09 (0.02)	0.05 (0.01)	0.08 (0.05)	0.06 (0.04)	0.20 (0.06)	0.21 (0.05)
18	0.04 (0.01)	0.31 (0.05)	0.19 (0.09)	0.18 (0.11)	0.04 (0.01)	0.17 (0.07)	0.31 (0.06)	0.29 (0.10)

## Data Availability

The original contributions presented in this study are included in the article/Supplementary Material. Further inquiries can be directed to the corresponding author.

## Supplementary Materials

The following supporting information can be downloaded at: https://www.mdpi.com/article/10.3390/biology15181671/s1, Supplementary S1: Figure S1: actograms of male C57BL/6 mice in DD conditions; Table S1: Individual *tau* of male mice under DD conditions (48 animals showed free-running behaviour and one animal did not contribute to ICC results); Table S2: Mean *tau* for male C57BL/6 mice kept in DD, averaged across seven days prior sacrifice; Supplementary S2: Table S1: F Values for ANOVA tests; Table S2: Normalised density of GABA_A_R α1, α5, β3, and γ2 subunits expression in male C57BL/6 mice kept in LD12:12; Table S3: Normalised density of GABA_A_R α1, α5, β3, and γ2 subunits expression in female C57BL/6 mice kept in LD12:12; Table S4: Normalised density of GABA_A_R α1, α5, β3, and γ2 subunit expression in male C57BL/6 mice kept in DD; Table S5: Mean *tau* for male C57BL/6 mice kept in DD, averaged across seven days prior sacrifice.
