# Supplementary material for: Temporal Variation and Region- and Sex-Specific Expression of GABAA Receptor Subunits in the Mouse Suprachiasmatic Nucleus
Source: Biology (Basel). 2026 Sep 21;15(18):1671. doi: 10.3390/biology15181671 (PMC13604189; doi:10.3390/biology15181671)
Supplement: Supplementary file 1 [file biology-15-01671-s001.zip › Supplementary S1.pdf]

Supplementary S1: Figure S1: actograms of male C57BL/6 mice in DD conditions

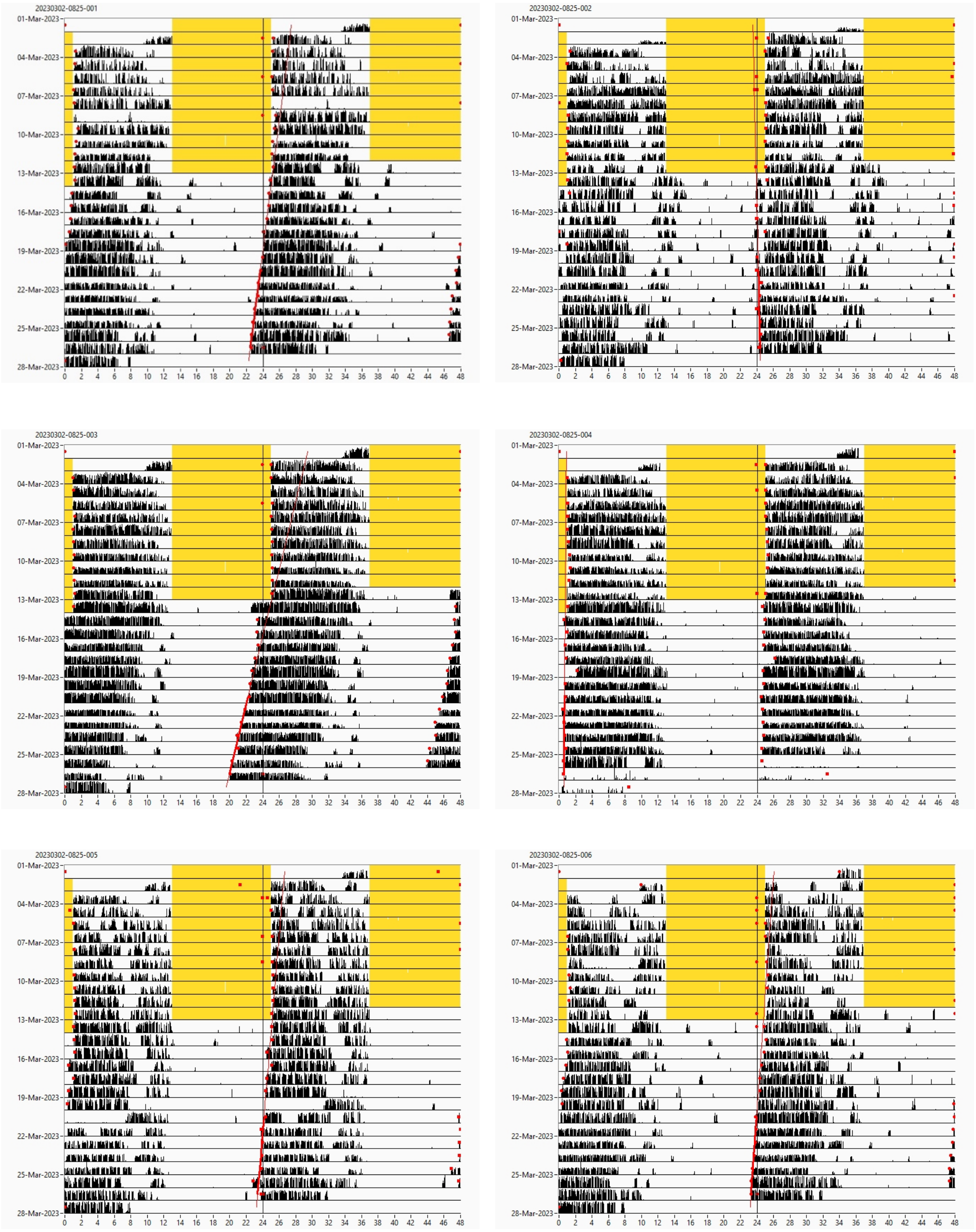

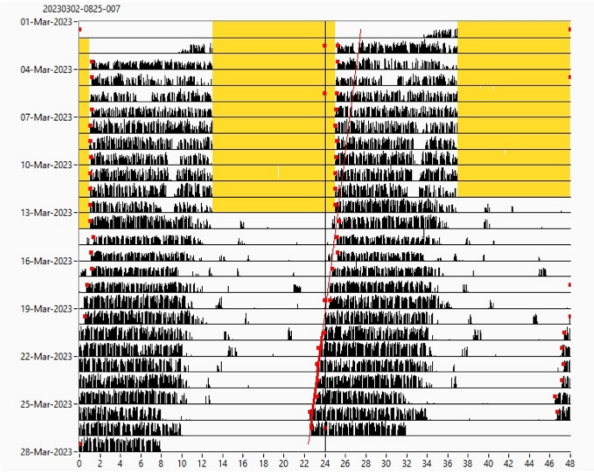
20230302-0825-007
01-Mar-2023
04-Mar-2023
07-Mar-2023
10-Mar-2023
13-Mar-2023
16-Mar-2023
19-Mar-2023
22-Mar-2023
25-Mar-2023
28-Mar-2023
0 2 4 6 8 10 12 14 16 18 20 22 24 26 28 30 32 34 36 38 40 42 44 46 48

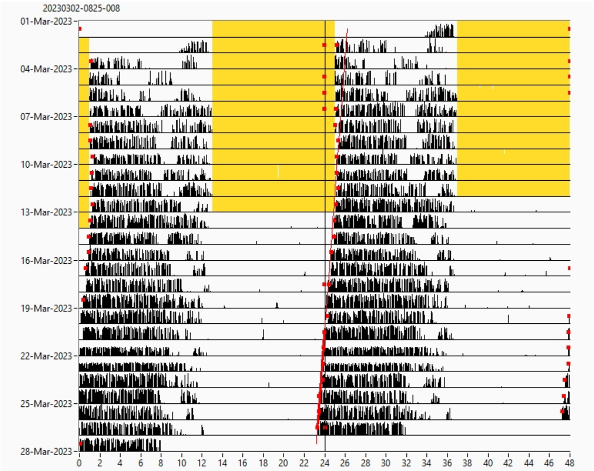
20230302-0825-008
01-Mar-2023
04-Mar-2023
07-Mar-2023
10-Mar-2023
13-Mar-2023
16-Mar-2023
19-Mar-2023
22-Mar-2023
25-Mar-2023
28-Mar-2023
0 2 4 6 8 10 12 14 16 18 20 22 24 26 28 30 32 34 36 38 40 42 44 46 48

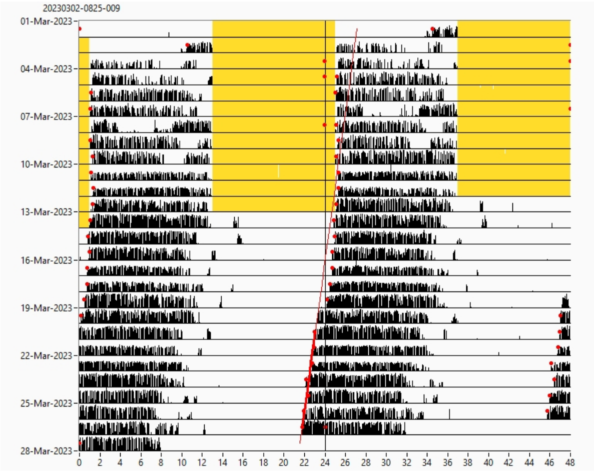
20230302-0825-009
01-Mar-2023
04-Mar-2023
07-Mar-2023
10-Mar-2023
13-Mar-2023
16-Mar-2023
19-Mar-2023
22-Mar-2023
25-Mar-2023
28-Mar-2023
0 2 4 6 8 10 12 14 16 18 20 22 24 26 28 30 32 34 36 38 40 42 44 46 48

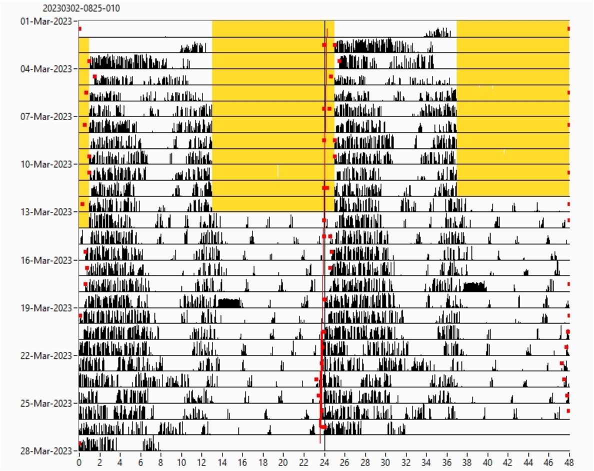
20230302-0825-010
01-Mar-2023
04-Mar-2023
07-Mar-2023
10-Mar-2023
13-Mar-2023
16-Mar-2023
19-Mar-2023
22-Mar-2023
25-Mar-2023
28-Mar-2023
0 2 4 6 8 10 12 14 16 18 20 22 24 26 28 30 32 34 36 38 40 42 44 46 48

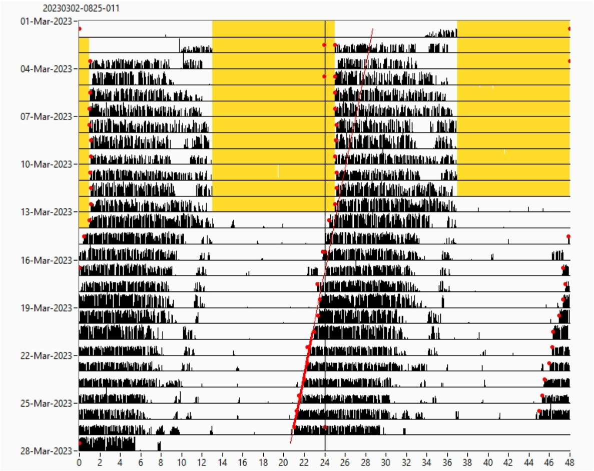
20230302-0825-011
01-Mar-2023
04-Mar-2023
07-Mar-2023
10-Mar-2023
13-Mar-2023
16-Mar-2023
19-Mar-2023
22-Mar-2023
25-Mar-2023
28-Mar-2023
0 2 4 6 8 10 12 14 16 18 20 22 24 26 28 30 32 34 36 38 40 42 44 46 48

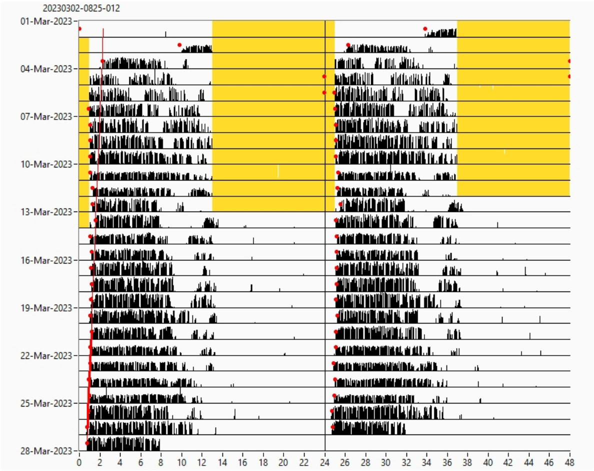
20230302-0825-012
01-Mar-2023
04-Mar-2023
07-Mar-2023
10-Mar-2023
13-Mar-2023
16-Mar-2023
19-Mar-2023
22-Mar-2023
25-Mar-2023
28-Mar-2023
0 2 4 6 8 10 12 14 16 18 20 22 24 26 28 30 32 34 36 38 40 42 44 46 48

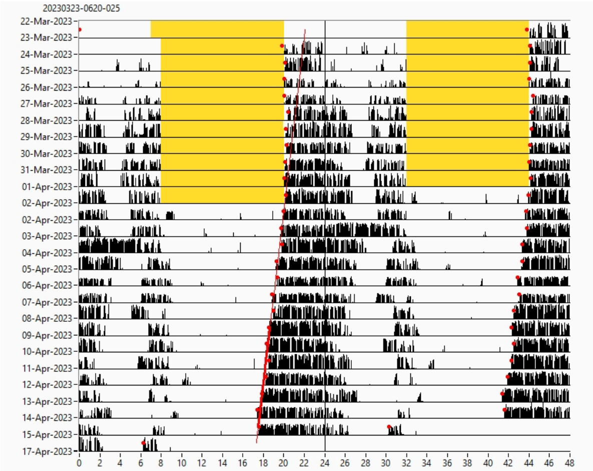
20230323-0620-025

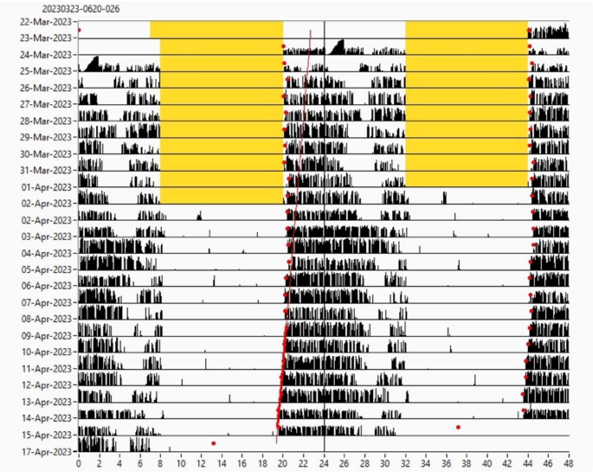
20230323-0620-026

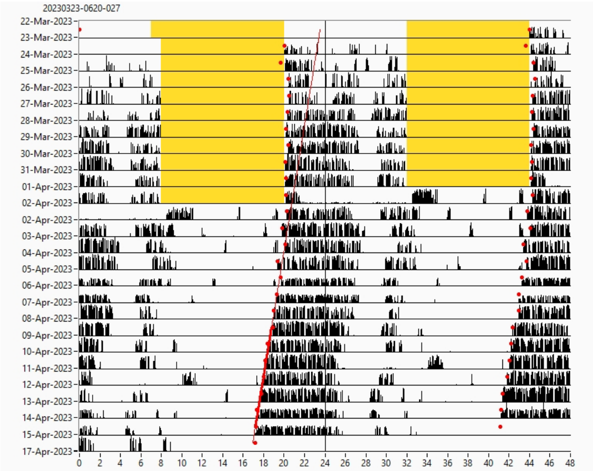
20230323-0620-027

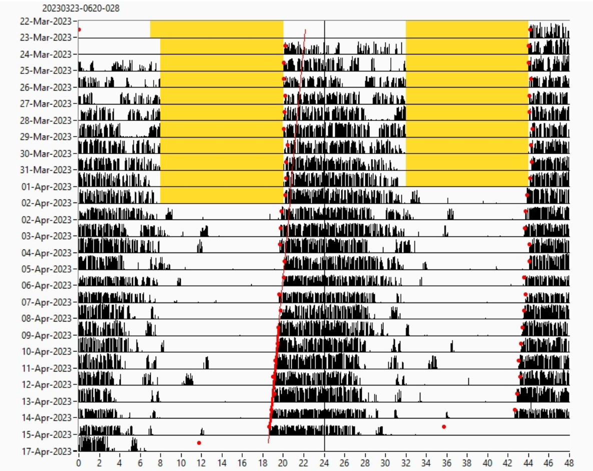
20230323-0620-028

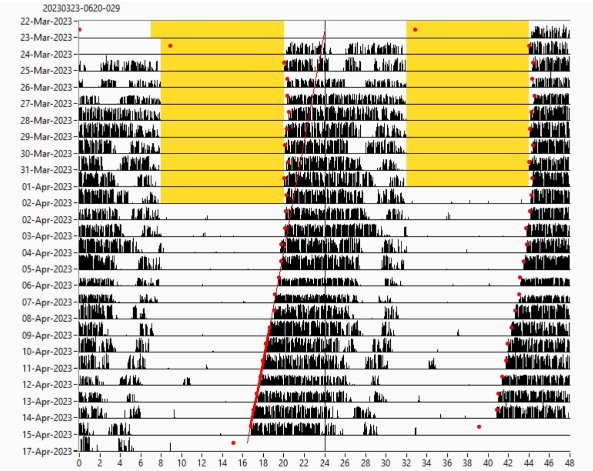
20230323-0620-029

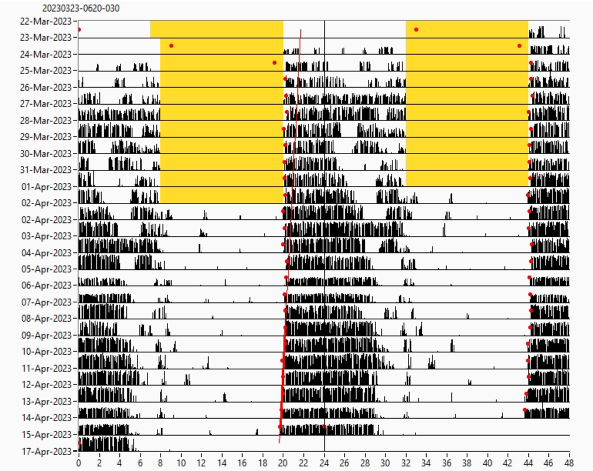
20230323-0620-030

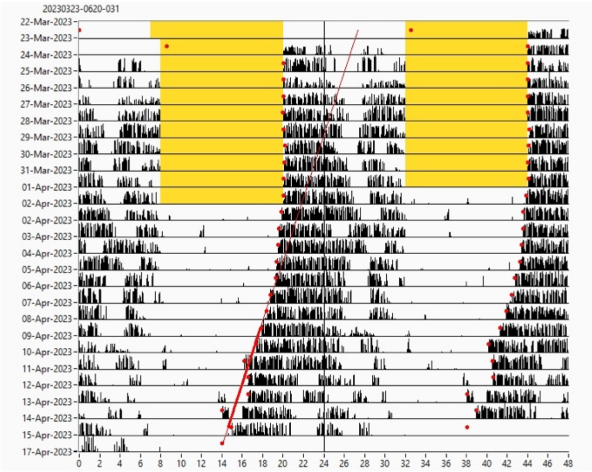
20230323-0620-031

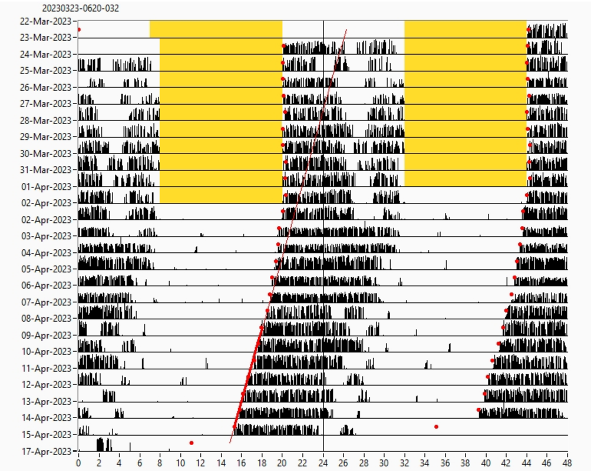
20230323-0620-032

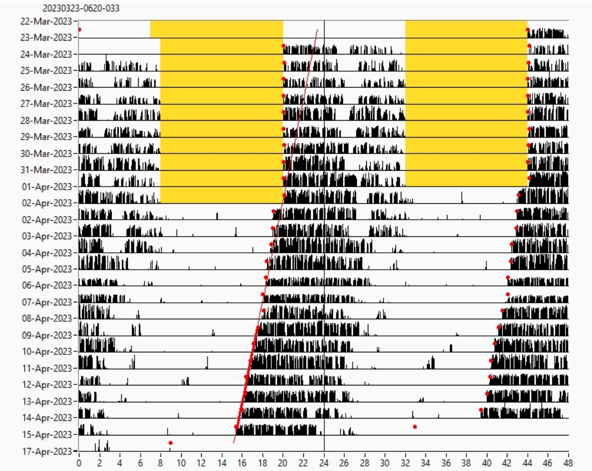
20230323-0620-033

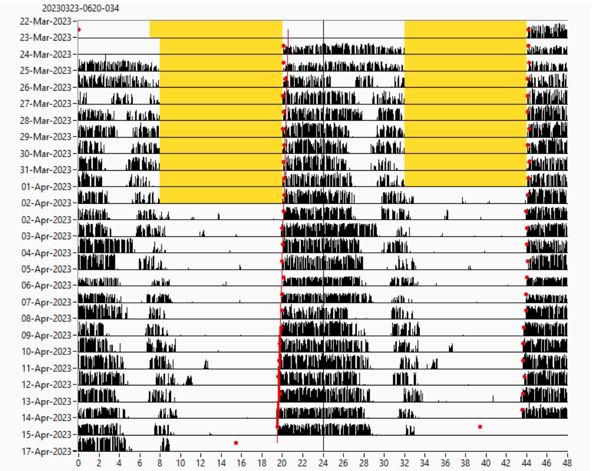
20230323-0620-034

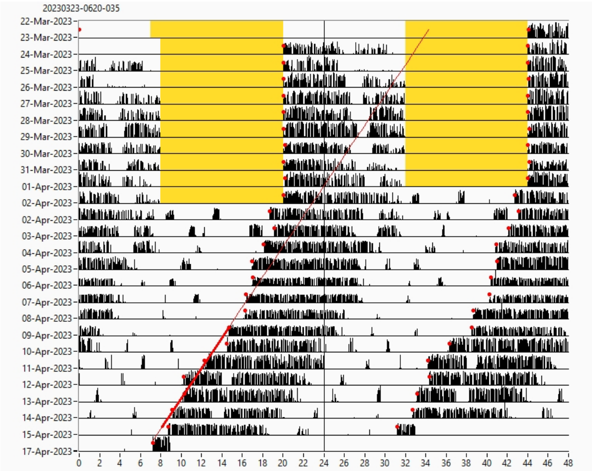
20230323-0620-035

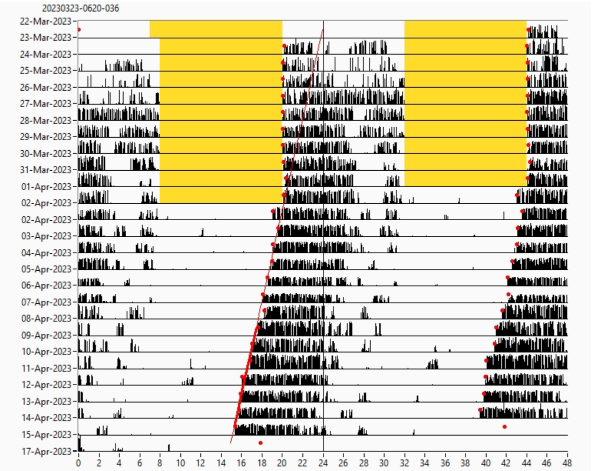
20230323-0620-036

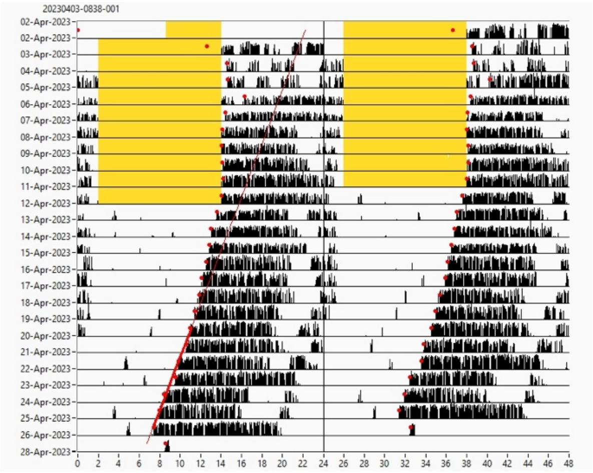
20230403-0838-001

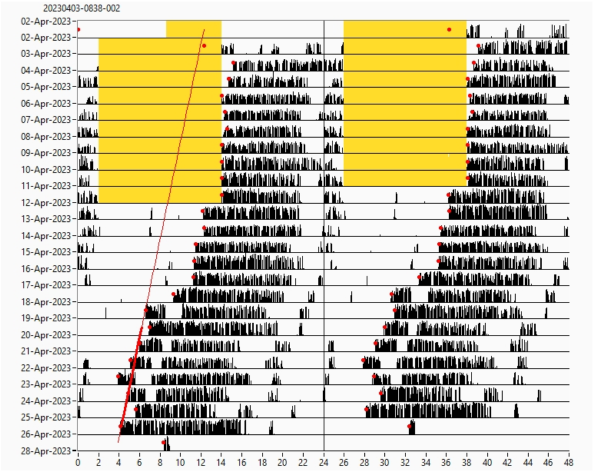
20230403-0838-002

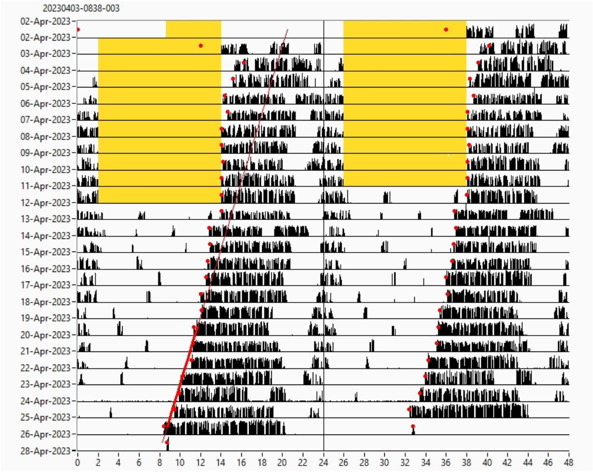
20230403-0838-003

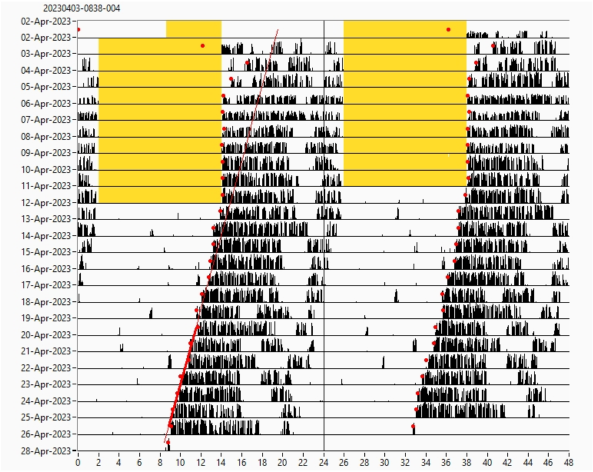
20230403-0838-004

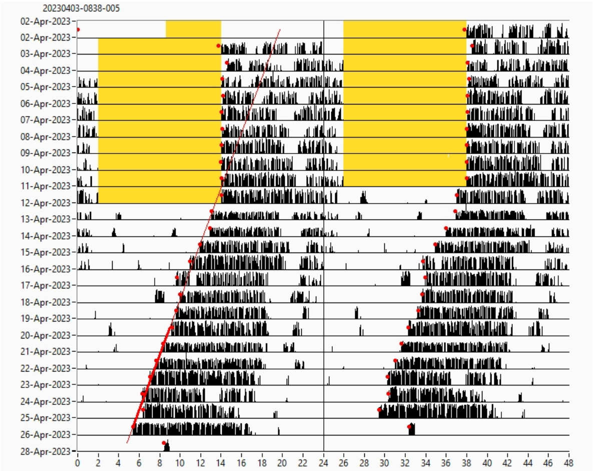
20230403-0838-005

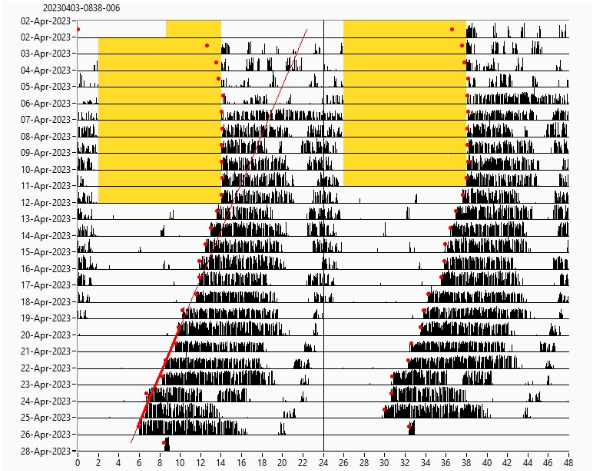
20230403-0838-006

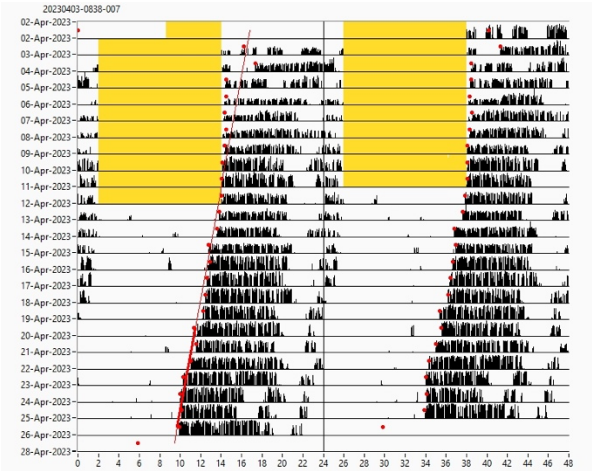
20230403-0838-007

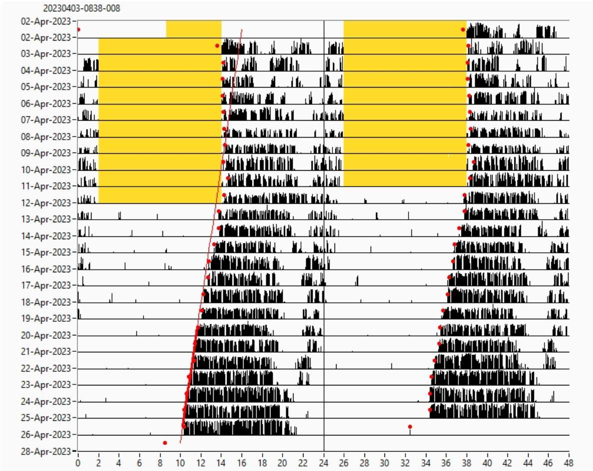
20230403-0838-008

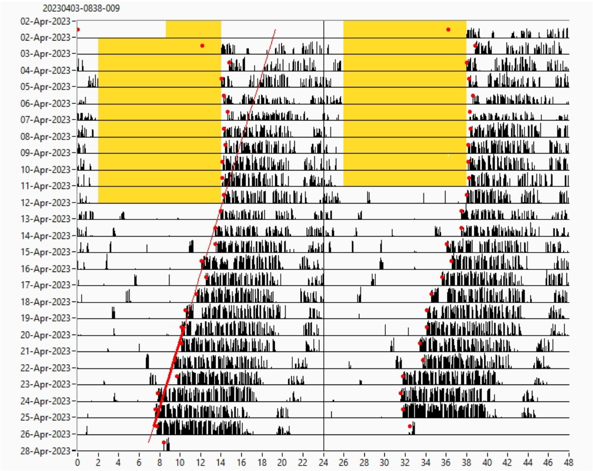
20230403-0838-009

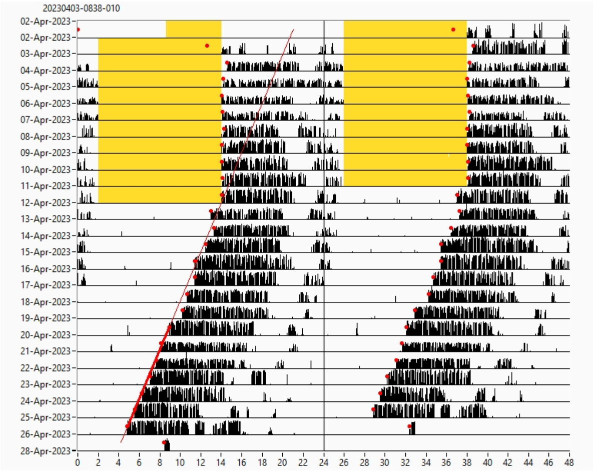
20230403-0838-010

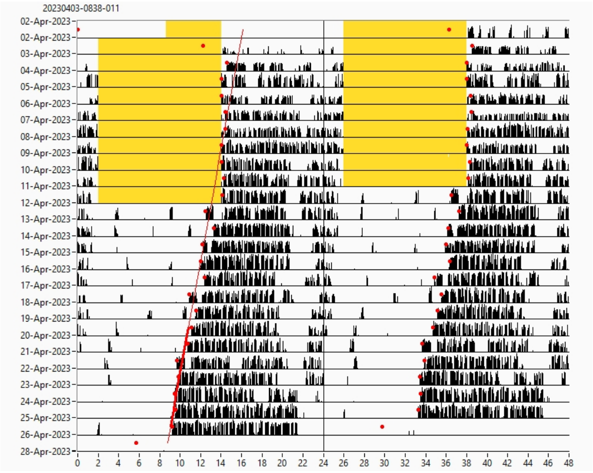
20230403-0838-011

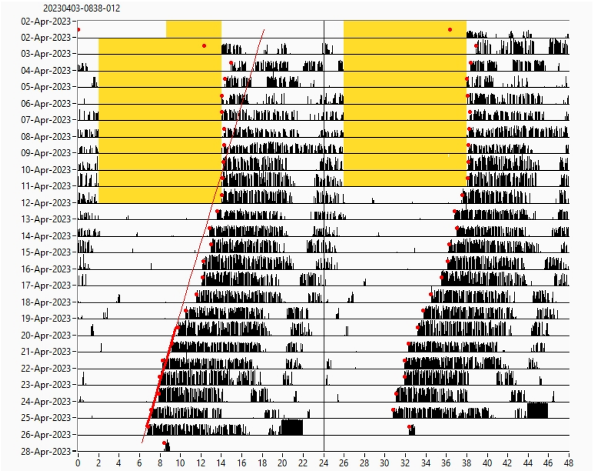
20230403-0838-012

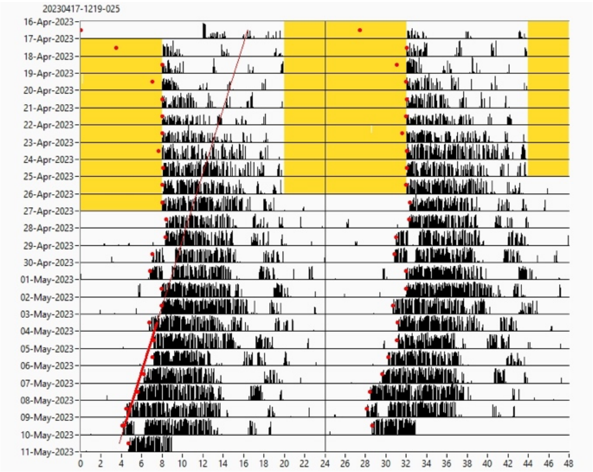
20230417-1219-025
16-Apr-2023 17-Apr-2023 18-Apr-2023 19-Apr-2023 20-Apr-2023 21-Apr-2023 22-Apr-2023 23-Apr-2023 24-Apr-2023 25-Apr-2023 26-Apr-2023 27-Apr-2023 28-Apr-2023 29-Apr-2023 30-Apr-2023 01-May-2023 02-May-2023 03-May-2023 04-May-2023 05-May-2023 06-May-2023 07-May-2023 08-May-2023 09-May-2023 10-May-2023 11-May-2023
0 2 4 6 8 10 12 14 16 18 20 22 24 26 28 30 32 34 36 38 40 42 44 46 48

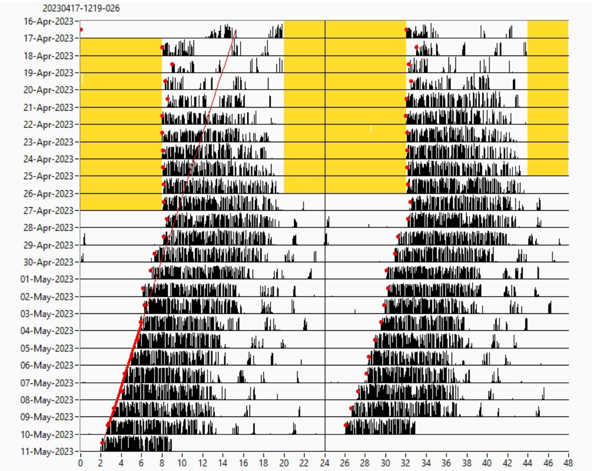
20230417-1219-026
16-Apr-2023 17-Apr-2023 18-Apr-2023 19-Apr-2023 20-Apr-2023 21-Apr-2023 22-Apr-2023 23-Apr-2023 24-Apr-2023 25-Apr-2023 26-Apr-2023 27-Apr-2023 28-Apr-2023 29-Apr-2023 30-Apr-2023 01-May-2023 02-May-2023 03-May-2023 04-May-2023 05-May-2023 06-May-2023 07-May-2023 08-May-2023 09-May-2023 10-May-2023 11-May-2023
0 2 4 6 8 10 12 14 16 18 20 22 24 26 28 30 32 34 36 38 40 42 44 46 48

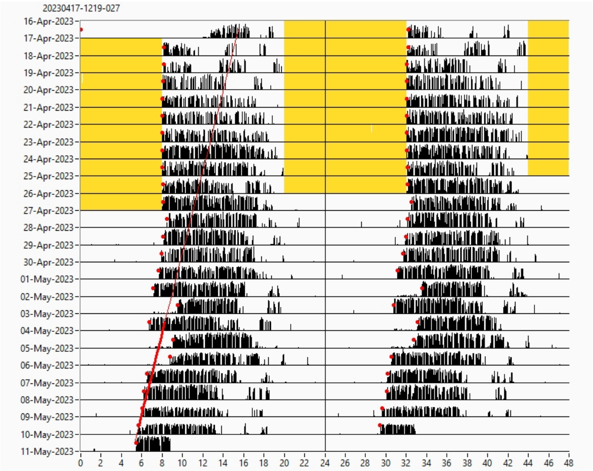
20230417-1219-027
16-Apr-2023 17-Apr-2023 18-Apr-2023 19-Apr-2023 20-Apr-2023 21-Apr-2023 22-Apr-2023 23-Apr-2023 24-Apr-2023 25-Apr-2023 26-Apr-2023 27-Apr-2023 28-Apr-2023 29-Apr-2023 30-Apr-2023 01-May-2023 02-May-2023 03-May-2023 04-May-2023 05-May-2023 06-May-2023 07-May-2023 08-May-2023 09-May-2023 10-May-2023 11-May-2023
0 2 4 6 8 10 12 14 16 18 20 22 24 26 28 30 32 34 36 38 40 42 44 46 48

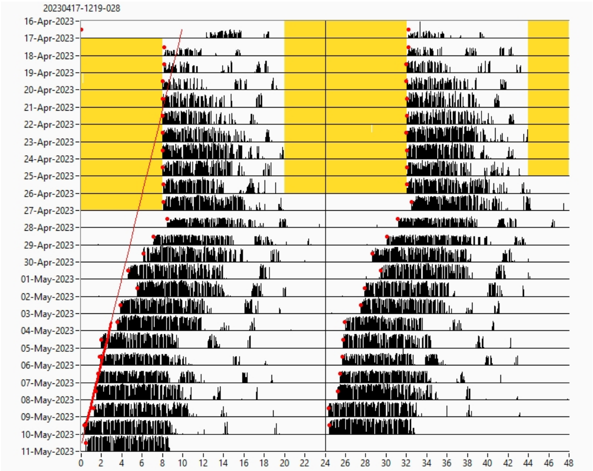
20230417-1219-028
16-Apr-2023 17-Apr-2023 18-Apr-2023 19-Apr-2023 20-Apr-2023 21-Apr-2023 22-Apr-2023 23-Apr-2023 24-Apr-2023 25-Apr-2023 26-Apr-2023 27-Apr-2023 28-Apr-2023 29-Apr-2023 30-Apr-2023 01-May-2023 02-May-2023 03-May-2023 04-May-2023 05-May-2023 06-May-2023 07-May-2023 08-May-2023 09-May-2023 10-May-2023 11-May-2023
0 2 4 6 8 10 12 14 16 18 20 22 24 26 28 30 32 34 36 38 40 42 44 46 48

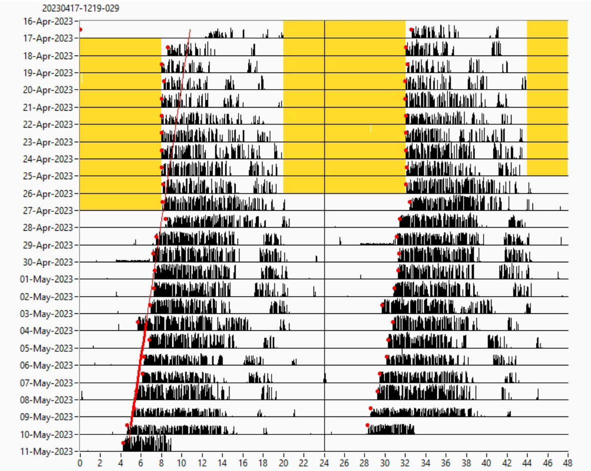
20230417-1219-029
16-Apr-2023 17-Apr-2023 18-Apr-2023 19-Apr-2023 20-Apr-2023 21-Apr-2023 22-Apr-2023 23-Apr-2023 24-Apr-2023 25-Apr-2023 26-Apr-2023 27-Apr-2023 28-Apr-2023 29-Apr-2023 30-Apr-2023 01-May-2023 02-May-2023 03-May-2023 04-May-2023 05-May-2023 06-May-2023 07-May-2023 08-May-2023 09-May-2023 10-May-2023 11-May-2023
0 2 4 6 8 10 12 14 16 18 20 22 24 26 28 30 32 34 36 38 40 42 44 46 48

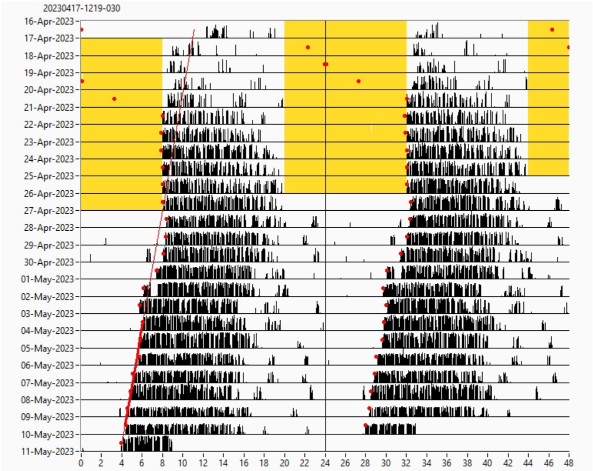
20230417-1219-030
16-Apr-2023 17-Apr-2023 18-Apr-2023 19-Apr-2023 20-Apr-2023 21-Apr-2023 22-Apr-2023 23-Apr-2023 24-Apr-2023 25-Apr-2023 26-Apr-2023 27-Apr-2023 28-Apr-2023 29-Apr-2023 30-Apr-2023 01-May-2023 02-May-2023 03-May-2023 04-May-2023 05-May-2023 06-May-2023 07-May-2023 08-May-2023 09-May-2023 10-May-2023 11-May-2023
0 2 4 6 8 10 12 14 16 18 20 22 24 26 28 30 32 34 36 38 40 42 44 46 48

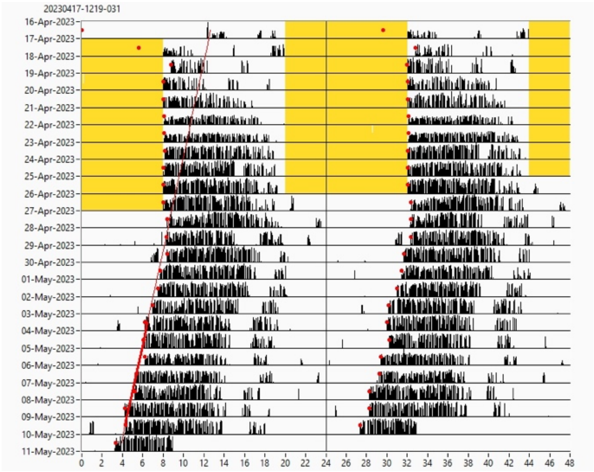
20230417-1219-031

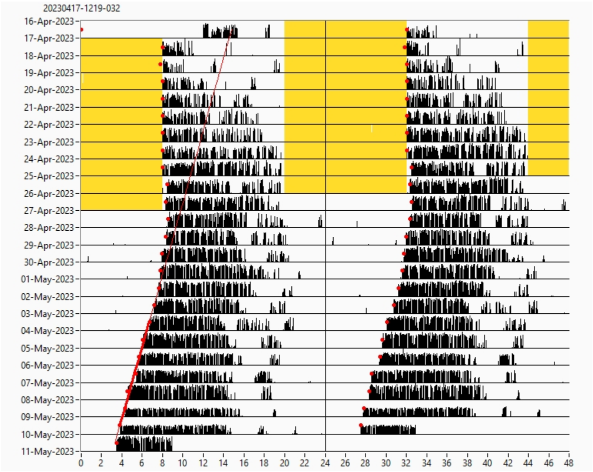
20230417-1219-032

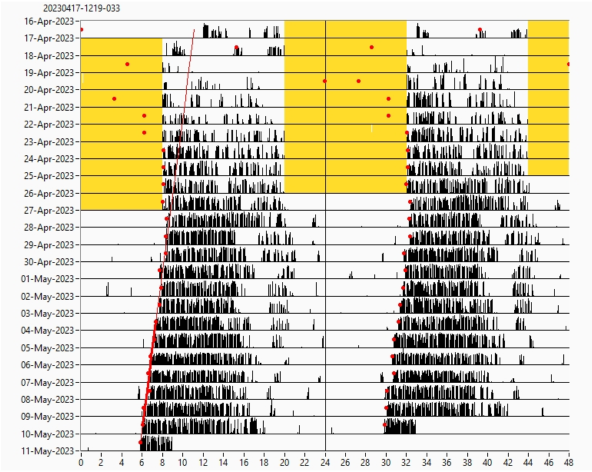
20230417-1219-033

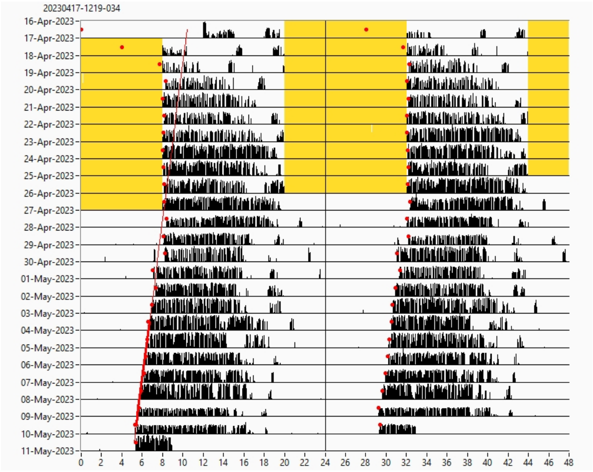
20230417-1219-034

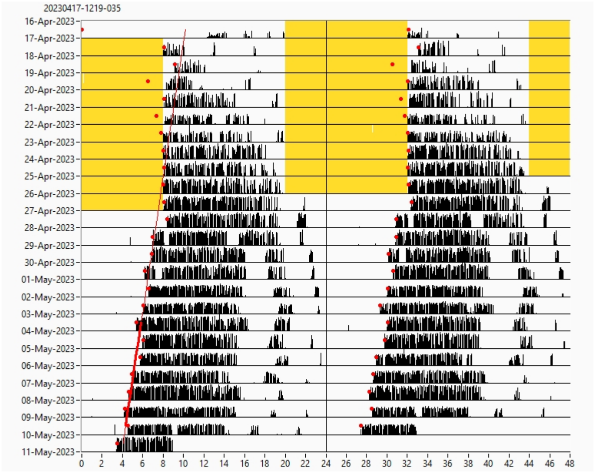
20230417-1219-035

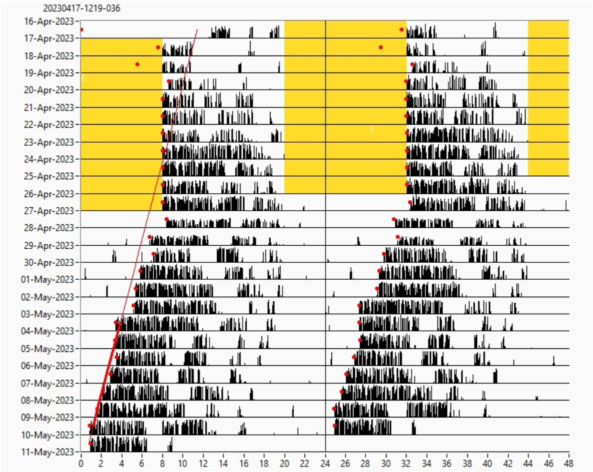
20230417-1219-036

Table S1: Individual *tau* of male mice under DD conditions (48 animals showed free-running behaviour and one animal did not contribute to ICC results)

Circadian time (CT)	Mean *tau* (τ)	SEM	n
**0**	23.84	0.031	12
**6**	23.69	0.080	12
**12**	23.53	0.044	12
**18**	23.65	0.036	12
**Overall**	23.68	-	48

Table S2 Mean *tau* for male C57BL/6 mice kept in DD, averaged across seven days prior sacrifice

Circadian time (CT)	Mouse	*Tau*
**CT0**	1.1.1	23.80
	1.2.2	23.94
	1.3.3	23.62
	1.4.4	24.00
	1.5.5	23.87
	1.6.6	23.89
	1.7.8	23.81
	1.8.9	23.89
	1.9.10	23.79
	1.10.11	23.84
	1.11.12	23.69
	1.12.13	23.94
**CT6**	3.25.29	23.81
	3.26.30	23.86
	3.27.31	23.74
	3.28.32	23.86
	3.29.33	23.70
	3.30.34	23.92

	3.31.36	23.61
	3.32.37	23.54
	3.33.38	23.67
	3.34.39	23.96
	3.35.40	22.91
	3.36.41	23.64
**CT12**	1.1.1	23.38
	1.2.2	23.66
	1.3.3	23.51
	1.4.4	23.56
	1.5.5	23.40
	1.6.6	23.31
	1.7.8	23.70
	1.8.9	23.76
	1.9.10	23.50
	1.10.11	23.33
	1.11.12	23.70
	1.12.13	23.52
**CT18**	3.25.29	23.48
	3.26.30	23.46
	3.27.31	23.81
	3.28.32	23.59
	3.29.33	23.75
	3.30.34	23.70
	3.31.36	23.64
	3.32.37	23.52
	3.33.38	23.78
	3.34.39	23.78
	3.35.40	23.74
	3.36.41	23.55
