# Supplementary material for: Temporal Variation and Region- and Sex-Specific Expression of GABAA Receptor Subunits in the Mouse Suprachiasmatic Nucleus
Source: Biology (Basel). 2026 Sep 21;15(18):1671. doi: 10.3390/biology15181671 (PMC13604189; doi:10.3390/biology15181671)
Supplement: Supplementary file 1 [file biology-15-01671-s001.zip › Supplementary S2.pdf]

Supplementary S2

Table S1: F Values for ANOVA tests

Set	n per ZT	ANOVA	Tukey: sig. pairs
LD Alpha1 males	5, 6, 5, 6	F(3,18) =7.33, p=0.0021	ZT12-ZT6; ZT18-ZT6
LD Alpha1 females	5, 6, 6, 3	F(3,16)=3.89, p=0.029	ZT18-ZT0
LD Alpha5 males	4, 5, 5, 6	F(3,16)=14.3, p=<0.001	ZT6-ZT0; ZT18-ZT6; ZT18-ZT12
LD Alpha5 females	5, 5, 6, 5	F(3,17)=38.48, p=<0.001	ZT6-ZT0; ZT12-ZT0; ZT18-ZT0
LD Beta3 males	2, 4, 4, 6	F(3,12)=3.93, p=0.036	none
LD Beta3 females	6, 6, 5, 6	F(3,19)=1.84, p=0.17	none
LD Gamma2 males	3, 4, 6, 6	F(3,15)=4.76, p=0.016	none
LD Gamma2 females	6, 6, 5, 6	F(3,19)=2.59, p=0.083	none
DD Alpha1 males	6, 6, 6, 6	F(3,20)=7.63, p=0.0014	ZT12-ZT0; ZT12-ZT6; ZT18-ZT6
DD Alpha5 males	5, 6, 6, 6	F(3,19)=1.92, p=0.16	none
DD Beta3 males	5, 6, 6, 6	F(3,19)=2.72, p=0.073	none
DD Gamma2 males	5, 6, 6, 6	F(3,19)=1.48, p=0.25	none

Table S2 Normalised density of $GABA_AR$ α1, α5, β3, and γ2 subunits expression in male C57BL/6 mice kept in LD12:12

Subunit	Zeitgeber time (ZT)	Mouse	Number of sections	Mean Normalised Density
α1	0	1	2	0.395714
		2	2	0.418509
		3	4	0.428425
		4	3	0.375937
		5	6	0.343564
	6	1	8	0.444994
		2	9	0.435956
		3	2	0.511794
		4	2	0.553145
		5	2	0.388929
		6	4	0.568568
	12	1	3	0.500051
		2	6	0.284750
		3	8	0.190980
		4	6	0.318237
		5	2	0.359976
	18	1	5	0.392400
		2	10	0.337806
		3	2	0.281465
		4	6	0.241289
		5	8	0.155208
		6	7	0.245920
α5	0	1	2	0.501882
		2	4	0.303389
		3	8	0.216917

		4	7	0.258097
	6	1	7	0.015904
		2	8	0.02615
		3	4	0.120879
		4	8	0.022001
		5	7	0.117647
	12	1	7	0.214948
		2	5	0.187284
		3	8	0.306095
		4	3	0.147098
		5	1	0.123829
	18	1	6	0.397599
		2	6	0.764838
		3	6	0.828386
		4	8	0.449189
		5	4	0.456415
		6	7	0.402548
β3	0	1	4	0.675578
		2	6	0.790519
	6	1	5	0.580157
		2	7	0.270416
		3	1	0.342226
		4	2	0.414963
	12	1	3	0.421763
		2	5	0.361394
		3	6	0.425193
		4	6	0.613871
	18	1	5	0.718147
		2	7	0.447812
		3	3	0.788084

		4	6	0.631286
		5	8	0.426030
		6	7	0.566579
**γ2**	**0**	1	3	0.696315
		2	4	0.667941
		3	6	0.742895
	**6**	1	5	0.617447
		2	7	0.466706
		3	1	0.415694
		4	2	0.381668
	**12**	1	3	0.424308
		2	5	0.443582
		3	6	0.227164
		4	1	0.407384
		5	6	0.698303
		6	2	0.678331
	**18**	1	6	0.641900
		2	7	0.790935
		3	3	0.730089
		4	6	0.635709
		5	8	0.627783
		6	7	0.632218

Table S3 Normalised density of $GABA_AR$ α1, α5, β3, and γ2 subunits expression in female C57BL/6 mice kept in LD12:12

Subunit	Zeitgeber time (ZT)	Mouse	Number of sections	Mean Normalised Density
α1	0	1	8	0.458059
		2	6	0.065354
		3	3	0.773563
		4	1	0.781139
		5	2	0.674981
	6	1	2	0.213328
		2	8	0.22379
		3	7	0.491291
		4	6	0.193005
		5	6	0.252878
		6	10	0.15515
	12	1	5	0.422272
		2	4	0.159274
		3	5	0.110711
		4	1	0.421381
		5	7	0.202141
		6	4	0.319259
	18	1	6	0.178911
		2	6	0.164791
		3	5	0.129700
α5	0	1	7	0.650543
		2	7	0.600633
		3	7	0.386032
		4	5	0.635595
		5	8	0.51269
	6	1	1	0.014264
		2	5	0.166594

		3	8	0.067951
		4	4	0.083550
		5	9	0.175665
	**12**	1	6	0.187072
		2	8	0.123953
		3	3	0.054807
		4	3	0.113514
		5	9	0.121011
		6	8	0.114291
	**18**	1	8	0.076721
		2	6	0.181443
		3	6	0.258691
		4	5	0.281323
		5	7	0.228835
**β3**	**0**	1	6	0.291591
		2	3	0.573955
		3	5	0.685343
		4	4	0.270214
		5	2	0.372409
		6	1	0.222171
	**6**	1	7	0.236645
		2	5	0.183735
		3	6	0.148883
		4	8	0.442345
		5	5	0.282999
		6	7	0.15564
	**12**	1	4	0.405350
		2	5	0.292389
		3	7	0.602242
		4	8	0.162013

		5	6	0.096344
	18	1	8	0.551403
		2	5	0.508621
		3	3	0.306623
		4	6	0.401195
		5	4	0.571157
		6	7	0.276267
γ2	0	1	6	0.564152
		2	3	0.588605
		3	5	0.673301
		4	4	0.709746
		5	2	0.160674
		6	1	0.656639
	6	1	7	0.731593
		2	5	0.763800
		3	6	0.599794
		4	8	0.750727
		5	5	0.675770
		6	7	0.693267
	12	1	4	0.531306
		2	6	0.653764
		3	7	0.376229
		4	8	0.644100
		5	6	0.294622
	18	1	6	0.611856
		2	4	0.585064
		3	7	0.668438
		4	8	0.782664
		5	5	0.690908
		6	3	0.6318337

Table S4 Normalised density of $GABA_AR$ α1, α5, β3, and γ2 subunit expression in male C57BL/6 mice kept in DD

Subunit	Circadian time (CT)	Mouse	Number of sections	Mean Normalised Density
**α1**	**0**	1	3	0.081804
		2	7	0.071064
		3	6	0.059156
		4	2	0.059084
		5	6	0.037437
		6	5	0.037957
	**6**	1	5	0.003105
		2	5	0.002656
		3	5	0.002588
		4	6	0.01745
		5	3	0.010755
		6	6	0.006031
	**12**	1	4	0.351955
		2	6	0.235104
		3	4	0.592166
		4	8	0.168825
		5	6	0.105109
		6	8	0.151072
	**18**	1	10	0.078112
		2	6	0.101769
		3	5	0.141798
		4	9	0.358509
		5	7	0.334627
		6	6	0.23243

**α5**	**0**	1	5	0.060992
		2	5	0.012634
		3	4	0.013824
		4	7	0.001848
		5	5	0.001836
	**6**	1	4	0.024876
		2	7	0.024952
		3	7	0.045677
		4	7	0.021158
		5	6	0.013822
		6	8	0.021853
	**12**	1	6	0.037388
		2	7	0.017955
		3	6	0.068024
		4	8	0.057735
		5	8	0.031513
		6	8	0.072135
	**18**	1	9	0.429854
		2	7	0.048215
		3	6	0.045365
		4	8	0.116068
		5	9	0.036107
		6	7	0.044972
**β3**	**0**	1	5	0.035243
		2	8	0.02153
		3	8	0.022953
		4	4	0.039701
		5	4	0.034843
	**6**	1	7	0.005159

		2	8	0.016059
		3	7	0.026155
		4	8	0.023798
		5	7	0.010818
		6	2	0.027094
	**12**	1	7	0.015277
		2	8	0.005227
		3	4	0.019077
		4	9	0.006871
		5	8	0.001229
		6	8	0.005374
	**18**	1	4	0.055417
		2	9	0.010265
		3	6	0.108546
		4	9	0.025178
		5	8	0.038637
		6	8	0.00587
**γ2**	**0**	1	3	0.242941
		2	9	0.017325
		3	7	0.133127
		4	4	0.071239
		5	4	0.194376
	**6**	1	8	0.175343
		2	7	0.325896
		3	6	0.409602
		4	6	0.162064
		5	4	0.115196
		6	6	0.166348

	12	1	4	0.231609
		2	7	0.25720
		3	9	0.402699
		4	9	0.207142
		5	9	0.156157
		6	8	0.052555
	18	1	9	0.228765
		2	6	0.656893
		3	6	0.191916
		4	9	0.224148
		5	7	0.159956
		6	5	0.349645

Table S5 Mean *tau* for male C57BL/6 mice kept in DD, averaged across seven days prior sacrifice

Circadian time (CT)	Mouse	*Tau*
CT0	1.1.1	23.80
	1.2.2	23.94
	1.3.3	23.62
	1.4.4	24.00
	1.5.5	23.87
	1.6.6	23.89
	1.7.8	23.81
	1.8.9	23.89
	1.9.10	23.79
	1.10.11	23.84
	1.11.12	23.69
	1.12.13	23.94
CT6	3.25.29	23.81
	3.26.30	23.86
	3.27.31	23.74
	3.28.32	23.86
	3.29.33	23.70
	3.30.34	23.92
	3.31.36	23.61
	3.32.37	23.54
	3.33.38	23.67
	3.34.39	23.96
	3.35.40	22.91
	3.36.41	23.64
CT12	1.1.1	23.38
	1.2.2	23.66
	1.3.3	23.51
	1.4.4	23.56

	1.5.5	23.40
	1.6.6	23.31
	1.7.8	23.70
	1.8.9	23.76
	1.9.10	23.50
	1.10.11	23.33
	1.11.12	23.70
	1.12.13	23.52
**CT18**	3.25.29	23.48
	3.26.30	23.46
	3.27.31	23.81
	3.28.32	23.59
	3.29.33	23.75
	3.30.34	23.70
	3.31.36	23.64
	3.32.37	23.52
	3.33.38	23.78
	3.34.39	23.78
	3.35.40	23.74
	3.36.41	23.55
